# RcSPL1–RcTAF15b regulates the flowering time of rose (*Rosa chinensis*)

**DOI:** 10.1093/hr/uhad083

**Published:** 2023-04-25

**Authors:** Rui Yu, Zhiying Xiong, Xinhui Zhu, Panpan Feng, Ziyi Hu, Rongxiang Fang, Yuman Zhang, Qinglin Liu

**Affiliations:** Department of Ornamental Horticulture, College of Horticulture, China Agricultural University, Beijing 100193, China; Department of Ornamental Horticulture, College of Horticulture, China Agricultural University, Beijing 100193, China; Department of Ornamental Horticulture, College of Horticulture, China Agricultural University, Beijing 100193, China; Department of Ornamental Horticulture, College of Horticulture, China Agricultural University, Beijing 100193, China; Department of Ornamental Horticulture, College of Horticulture, China Agricultural University, Beijing 100193, China; National Key Laboratory of Plant Genomics, Institute of Microbiology, Chinese Academy of Sciences, and National Plant Gene Research Center, Beijing 100101, China; University of Chinese Academy of Sciences, Beijing 101408, China; National Key Laboratory of Plant Genomics, Institute of Microbiology, Chinese Academy of Sciences, and National Plant Gene Research Center, Beijing 100101, China; Department of Ornamental Horticulture, College of Horticulture, China Agricultural University, Beijing 100193, China

## Abstract

Rose (*Rosa chinensis*), which is an economically valuable floral species worldwide, has three types, namely once-flowering (OF), occasional or re-blooming (OR), and recurrent or continuous flowering (CF). However, the mechanism underlying the effect of the age pathway on the duration of the CF or OF juvenile phase is largely unknown. In this study, we observed that the *RcSPL1* transcript levels were substantially upregulated during the floral development period in CF and OF plants. Additionally, accumulation of RcSPL1 protein was controlled by rch-miR156. The ectopic expression of *RcSPL1* in *Arabidopsis thaliana* accelerated the vegetative phase transition and flowering. Furthermore, the transient overexpression of *RcSPL1* in rose plants accelerated flowering, whereas silencing of *RcSPL1* had the opposite phenotype. Accordingly, the transcription levels of floral meristem identity genes (*APETALA1*, *FRUITFULL*, and *LEAFY*) were significantly affected by the changes in *RcSPL1* expression. RcTAF15b protein, which is an autonomous pathway protein, was revealed to interact with RcSPL1. The silencing and overexpression of *RcTAF15b* in rose plants led to delayed and accelerated flowering, respectively. Collectively, the study findings imply that RcSPL1–RcTAF15b modulates the flowering time of rose plants.

## Introduction

Rose (*Rosa chinensis*), which is known as the queen of flowers, is an economically and culturally important plant species. Rose plants are cultivated worldwide as garden plants or for the production of cut flowers [1]. According to their flowering habits, rose plants have been classified into the following three types: recurrent or continuous flowering (CF) (flowers bloom continuously under appropriate conditions); occasional re-blooming (OR) genotype (flowers bloom in spring and occasionally in autumn); and once-flowering (OF) (flowers bloom only in spring) [2]. Previous studies on the molecular basis of CF behavior mainly focused on the gibberellin (GA) pathway as well as on the floral repressor gene *TERMINAL FLOWER 1* (*TFL1*) [2–8]. However, CF seedlings have a short juvenile phase (4–5 weeks) with a determinate growth pattern, whereas OF seedlings have a long juvenile phase (1–3 years) with an indeterminate growth pattern [9, 10]. Therefore, we hypothesized that the flowering-related age pathway may contribute to the differences in floral development among rose types.

In plants, flowering at the appropriate time is critical for reproductive success. Additionally, the transition to the flowering stage is regulated by the complex interplay between environmental cues and genetic pathways [11, 12]. Genetic analyses revealed six flowering response pathways (age, vernalization, photoperiod, GA, autonomous, and ambient temperature pathways). Genes encoding flowering time integrators, including *APETALA 1* (*AP1*), *SUPPRESSOR OF OVEREXPRESSION OF CO1* (*SOC1*), *FLOWERING LOCUS T* (*FT*), and *LEAFY* (*LFY*), are integrated into these flowering pathways [12–14].

The age pathway is important for the transition from vegetative growth to reproductive development and flowering under non-inductive conditions [15]. In this pathway, microRNA156 (miR156) and its target *SQUAMOSA PROMOTER BINDING-LIKE* (*SPL*) genes are the key components [16]. Earlier research confirmed that miRNAs are non-coding small RNAs (20–24 nt) that are essential for post-transcriptional gene regulation in plants because they cleave target transcripts and/or inhibit translation [17, 18]. Moreover, the miR156 family members comprise typically conserved miRNAs that target most *SPL* genes in various species [15, 19].

The *SQUAMOSA* promoter-binding proteins (SBPs) were initially identified as transcriptional regulators that bind to the promoter of the floral meristem identity gene in *Antirrhinum majus* [20]. In addition, SPLs are characterized by an SBP domain (76–79 amino acids) that includes a nuclear localization signal and two zinc (Zn)-finger-like structural motifs [21]. The SPLs are encoded by multigene families in *Arabidopsis thaliana*, rice, tomato, grape, and maize (16, 19, 15, 18, and 31 members, respectively) and are functionally diverse proteins [22–25]. For example, in *A. thaliana*, SPL3, SPL4, and SPL5 contribute to changes in floral meristem identity, whereas SPL2, SPL9, SPL10, SPL11, SPL13, and SPL15 help mediate the juvenile-to-adult phase transition, while also inducing the vegetative-to-reproductive stage transition [26]. The *SPL* genes identified to date reportedly encode proteins that affect plant architecture as well as diverse development-related processes, including phase transitions, GA biosynthesis and signaling, and somatic embryogenesis [27–32]. However, the functional characterization of rose *SPL* genes has lagged behind that of *SPL* genes from other plant species because of an insufficient amount of genomic information and the problems associated with rose regeneration.

The autonomous pathway promotes flowering independently of day length. Several genes, including *TATA-BINDING PROTEIN-ASSOCIATED FACTOR 15b* (*TAF15b*), *FLOWERING LOCUS KH DOMAIN* (*FLK*), *FLOWERING LOCUS CA* (FCA), *FLOWERING LOCUS PA* (*FPA*), and *FLOWERING LOCUS VE* (*FVE*), have been identified as crucial factors regulating floral development in this pathway. Among the encoded proteins, TAF15b is a component of the transcription factor IID complex (TFIID) with a Zn-finger motif and an RNA-recognition motif [33]. Although 18 putative *TAF* genes in *A. thaliana* have been analyzed, with some of them encoding proteins influencing various developmental processes, little is known about the TAF functions related to floral development [34–37].

**Figure 1:**
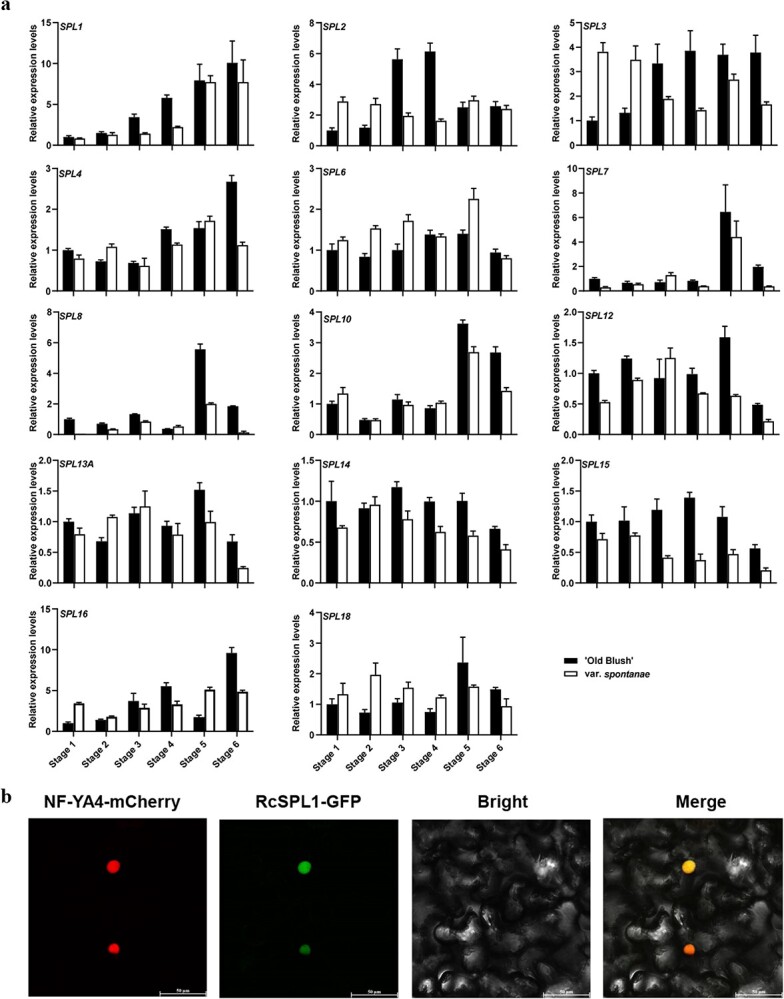
Characterization analysis of *RcSPL1*. **a** Expression profiles of 14 *SPL* genes in ‘Old Blush’ and *R. chinensis* var. *spontanea* during flower development. Stage 1, earliest bud break; stage 2, shoot elongation (>20 mm) without three-leaflet leaf; stage 3, the first three-leaflet leaf is visible; stage 4, the first five-leaflet leaf is visible; stage 5, flower bud appearance; stage 6, flower bud with a diameter of 5–7 mm. Mean values ± standard deviation are shown from three biological replicates (*n* = 3). *RcUBI2* was used as the internal control. **b** Subcellular localization of RcSPL1-GFP heterologously expressed in *N. benthamiana* leaves. The plasmid pSuper:*RcSPL1-*GFP was co-infiltrated with the nuclear marker pSuper:*NF-YA4*-mCherry. Green and red fluorescences were visualized by confocal microscope (Leica TCS SP8) 3 days after infiltration. Scale bar, 50 μm*.* The experiment was performed independently three times, and representative results are shown.

Despite the ornamental and economic value of rose, genes that regulate flowering, especially those associated with CF and OF, remain relatively unexplored. *Rosa chinensis* ‘Old Blush’ and *R. chinensis* var. *spontanea*, which respectively exhibit CF and OF behaviors, are typically selected as the research materials for investigating flowering. In the present study, 15 *SPL* genes were identified in these two varieties and their expression patterns were examined during flower developmental stages. Among the analyzed genes, *RcSPL1* expression increased significantly in both varieties. Thus, *RcSPL1* was functionally characterized using transgenic *A. thaliana,* and rose in which the target gene was silenced or overexpressed via *Tobacco rattle virus *(TRV)-mediated transient expression. We also determined that RcTAF15b is an SPL1-interacting protein that helps control flowering time. These findings provide substantial insights into the mechanisms underlying rose floral development.

## Results

### *RcSPL1* expression increased continuously in rose flower developmental stages

Fifteen *SPL* genes were identified in the ‘Old Blush’ (https://lipm-browsers.toulouse.inra.fr/pub/RchiOBHm-V2/) and *R. chinensis* var. *spontanea* (https://ngdc.cncb.ac.cn/search/?dbId=gwh&q=GWHAAAF00000000&page=1) genome databases (Supplementary Data Table S1). Their expression patterns were examined during flower developmental stages in ‘Old Blush’ and *R. chinensis* var. *spontanea*. In this study, the rose floral development period was divided into six stages as previously described, with minor modifications [38]. In addition, internal morphology was determined using paraffin sections and scanning electron microscopy (Supplementary Data Fig. S1, Supplementary Data Table S2). Reverse transcription–quantitative PCR (RT–qPCR) data indicated that, among the identified genes, the *SPL1* expression levels continuously increased as flowers developed in both ‘Old Blush’ and *R. chinensis* var. *spontanea* (Fig. 1a). Moreover, at each stage, the expression level was higher in ‘Old Blush’ than in *R. chinensis* var. *spontanea* (Fig. 1a), indicating that *SPL1* is crucial for the development of rose flowers. The expression patterns of the other *SPL* genes were less distinctive. Furthermore, *SPL9* expression was undetectable in both varieties. Accordingly, we focused on *SPL1* for the subsequent analysis. Notably, the *SPL1* sequence was the same in both varieties. Therefore, it was designated as *RcSPL1* in this study.

To evaluate the evolutionary relationship between *RcSPL1* and its orthologs in *A. thaliana*, the deduced amino acid sequences of the *SPL* genes were aligned using MEGA X for phylogenetic analysis, which showed that the RcSPL1 sequence was highly similar to AtSPL1 in *A. thaliana* (Supplementary Data Fig. S2a). The sequence alignment suggested that the SBP domain was highly conserved in RcSPL1 and FvSPL1 in woodland strawberry (*Fragaria vesca*) (Supplementary Data Fig. S2b). The sequence analysis also revealed a conserved nuclear localization signal and two conserved Zn-binding sites in the SBP domain (Supplementary Data Fig. S2c). The first and second Zn-finger structures were CCCH-type and CCHC-type, respectively (Supplementary Data Fig. S2c). These SBP domain structural characteristics are consistent with those in most other plants, implying that the SBP domain is highly conserved. Subcellular localization analysis indicated that the RcSPL1-GFP signal overlapped the mCherry signal (a nuclear marker) [39], providing evidence that RcSPL1 is a nuclear protein (Fig. 1b). The nuclear localization of RcSPL1 along with its increased expression during the flowering process suggests that RcSPL1 might be essential for rose floral development.

### *RcSPL1* expression was directly regulated by rch-miR156

**Figure 2:**
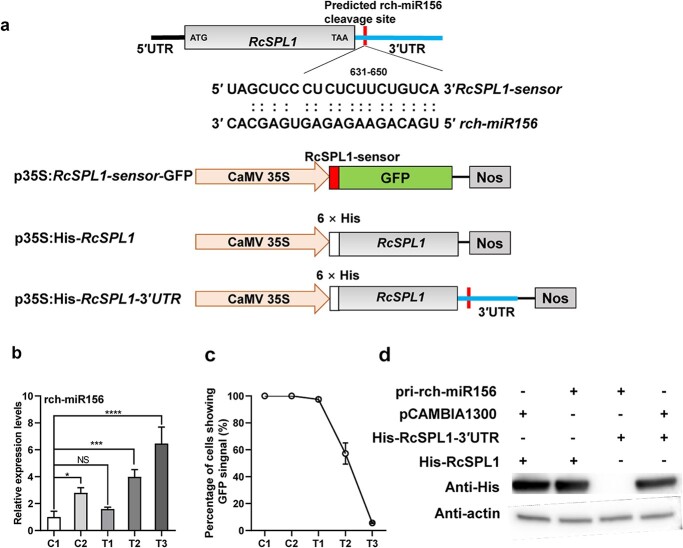
Targeting of RcSPL1 by rch-miR156 *in vivo*. **a** Sequence alignment of rch-miR156 complementary sequences of *RcSPL1* and schematic diagrams of constructions used to determine the regulation of cleavage of *RcSPL1* by rch-miR156. Upper, sequence alignment of rch-miR156 complementary sequences of *RcSPL1*. The rch-miR156-targeted site of *RcSPL1* is indicated in red. Middle and bottom, schematic diagrams of p35S:*RcSPL1-sensor*-GFP (RcSPL1-sensor-GFP), p35S:His-*RcSPL1 *(His-RcSPL1),  and p35S:His-*RcSPL1-3′UTR* (His-RcSPL1–3′UTR). **b** Transcript abundance of rch-miR156 in the leaves of *N. benthamiana* with different treatment. T1–T3, RcSPL1-sensor-GFP (OD_600_ = 0.5) was transiently co-expressed separately with different concentrations of p35S:*pri-rch-miR156* (pri-rch-miR156, OD_600_ = 0.2, 0.5, and 0.8) in the leaves of *N. benthamiana*. Cultures of pCAMBIA1300 + RcSPL1-sensor-GFP (C1) and pri-rch-miR156 + 130-GFP (C2) were used as controls, with OD_600_ = 0.5. Mean values ± standard deviation are shown from three biological replicates (*n* = 3). *NbU6* was used as the internal control. Asterisks represent statistically significant differences determined by Student’s *t-*test (^*^P < 0.05, ^***^P < 0.001, ^****^P < 0.0001). **c** Percentage of cells showing GFP signal in the same-size microscopic field. Mean values ± standard deviation are shown from five microscopic fields (*n* = 5). The experiments were performed independently three times, with similar results, and one representative result is shown. **d** His-RcSPL1–3′UTR and pri-rch-miR156 constructs transiently co-expressed in the leaves of *N. benthamiana*. Cultures of pCAMBIA1300 + His-RcSPL1, pri-rch-miR156 + His-RcSPL1, and pCAMBIA1300 + His-RcSPL1–3′UTR were used as controls, respectively. The experiments were performed independently three times with similar results.

MicroRNA156 was identified as a master regulator of flowering transition. Most previously reported miR156 family members negatively regulate *SPL* expression by directly cleaving *SPL* transcripts. The psRNATarget online tool predicted that *RcSPL1* is targeted by rch-miR156, with a potential cleavage site in the 3′ untranslated region (UTR) (Fig. 2a). Moreover, rch-MIR156 formed a local stem–loop structure and its sequence was highly similar to that of fve-MIR156e and ppe-MIR156e from *F. vesca* and *Prunus persica* (Supplementary Data Fig. S3a and b). To confirm the regulatory effect of rch-miR156 on *RcSPL1* expression, a green fluorescent protein (GFP) reporter assay was performed to monitor the cleavage of *RcSPL1* by rch-miR156 in a *Nicotiana benthamiana* transient expression system [40]. To compensate for the varying cleavage efficiency of rch-miR156, RcSPL1-sensor-GFP was co-expressed with different pri-rch-miR156 content in *N. benthamiana* (T1, T2, and T3). As controls, RcSPL1-sensor-GFP was co-expressed with the pCAMBIA1300 vector (C1) and pri-rch-miR156 was co-expressed with the 130-GFP vector (C2) (Fig. 2a). RT–qPCR analysis showed that rch-miR156 expression increased significantly as the pri-rch-miR156 content increased (Fig. 2b). Examination of cells in the same-size microscopic field detected a GFP signal in 100% of the cells in the control (C1 and C2) tobacco leaves (Fig. 2c). However, the percentage of cells with GFP signal decreased significantly as the expression of rch-miR156 increased, but there was no significant difference between T1 and C1 (Fig. 2c). In addition, the GFP fluorescence intensity in *N. benthamiana* leaves was consistent with the above-mentioned results (Supplementary Data Fig. S4). Hence, rch-miR156 downregulated *RcSPL1* expression, but only when it accumulated to a certain level. Additionally, the protein produced by the His-RcSPL1-3′UTR construct accumulated to significantly lower levels when it was co-expressed with pri-rch-miR156 than when it was co-expressed with the empty vector (pCAMBIA1300) or His-RcSPL1 co-expressed with pri-rch-miR156 in the *N. benthamiana* transient expression system (Fig. 2a and d, Supplementary Data Fig. S5). These results indicated that rch-miR156 effectively cleaved *RcSPL1* to directly regulate *RcSPL1* expression.

### Ectopic expression of *RcSPL1* in *A. thaliana* accelerated vegetative phase transition and flowering

**Figure 3:**
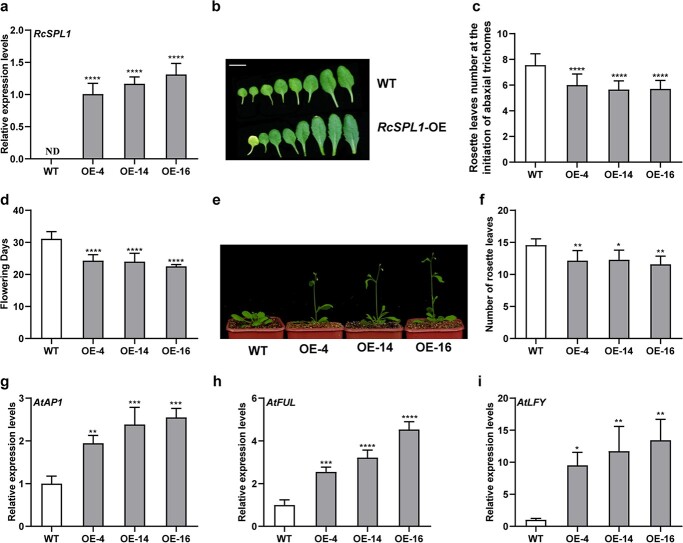
Ectopic expression of *RcSPL1* in WT *Arabidopsis*. **a** RT–qPCR analyses of *RcSPL1* transcript levels in *RcSPL1*-OE lines. OE-4, -14 and -16 correspond to three independent 35S:*RcSPL1* lines. Mean values ± standard deviation are shown from three biological replicates (*n* = 3). Asterisks represent statistically significant differences determined by Student’s *t-*test (^****^P < 0.0001). ND, not detected. *AtActin* was used as an internal control. **b** Morphology of rosette leaves of WT and *RcSPL1*-OE lines. Adult leaves were defined as those with abaxial trichomes and serrated leaf margins. Scal bar, 1 cm. **c** Rosette leaf number at the initation of abaxial trichomes of WT and *RcSPL1*-OE lines. Mean values ± standard deviation are from 13 plants. Asterisks represent statistically significant differences determined by Student’s *t-*test (^****^P < 0.0001). **d** Days to flowering of WT and transgenic plants. Mean values ± standard deviation are shown from 30 plants (*n* = 30). Asterisks represent statistically significant differences determined by Student’s *t-*test (****P < 0.0001). **e** Flowering phenotype comparison between WT and transgenic *Arabidopsis*. **f** Rosette leaf number of WT and *RcSPL1*-OE lines at flowering time. Mean values ± standard deviation are shown from 30 plants (*n* = 30). Asterisks represent statistically significant differences determined by Student’s *t-*test (^*^P < 0.05, ^**^P < 0.01). **g**–**i** RT–qPCR analyses of *AtAP1* (**g**), *AtFUL* (**h**), and *AtLFY* (**i**) transcript levels in *RcSPL1*-OE lines. Mean values ± standard deviation are shown from three biological replicates (*n* = 3). Asterisks represent statistically significant differences determined by Student’s *t-*test (^*^P < 0.05, ^**^P < 0.01, ^***^P < 0.001, ^****^P < 0.0001). *AtActin* was an internal control.

To assess whether RcSPL1 helps regulate floral development, *RcSPL1* was overexpressed in *A. thaliana*. Twenty-one *T*_2_ transgenic *A. thaliana* lines (*RcSPL1*-OE) with increased *RcSPL1* expression levels were obtained. Three *RcSPL1*-OE lines (OE-4, OE-14, and OE-16) were selected for functional characterization. RT–qPCR analysis involving *RcSPL1-*specific primers confirmed that *RcSPL1* expression levels were considerably higher in the *RcSPL1*-OE lines than in the wild-type (WT) plants (Fig. 3a). In *A. thaliana*, the production of trichomes on the abaxial surface of the leaf and the formation of a serrated leaf margin have been used to mark the vegetative phase transition (juvenile-to-adult transition) [41, 42]. To investigate whether *RcSPL1* influences the vegetative phase transition, WT plants and *RcSPL1*-OE lines were compared regarding their leaf morphology. Compared with the WT plants, the *RcSPL1*-OE plants greatly promoted the emergence of abaxial trichomes and serration on the leaf margin (Fig. 3b and c, Supplementary Data Table S3), indicating that *RcSPL1* accelerated the vegetative phase transition. Additionally, the *RcSPL1*-OE lines flowered 7–9 days earlier than the WT plants (Fig. 3d and e, Supplementary Data Table S3). Moreover, compared with the WT plants, the *RcSPL1-*OE lines had significantly fewer rosette leaves (Fig. 3f, Supplementary Data Table S3). Accordingly, the overexpression of *RcSPL1* in *A. thaliana* significantly promoted the vegetative phase transition and flowering.

### *RcSPL1-*silenced and -overexpressed rose plants had opposite flowering phenotypes

**Figure 4:**
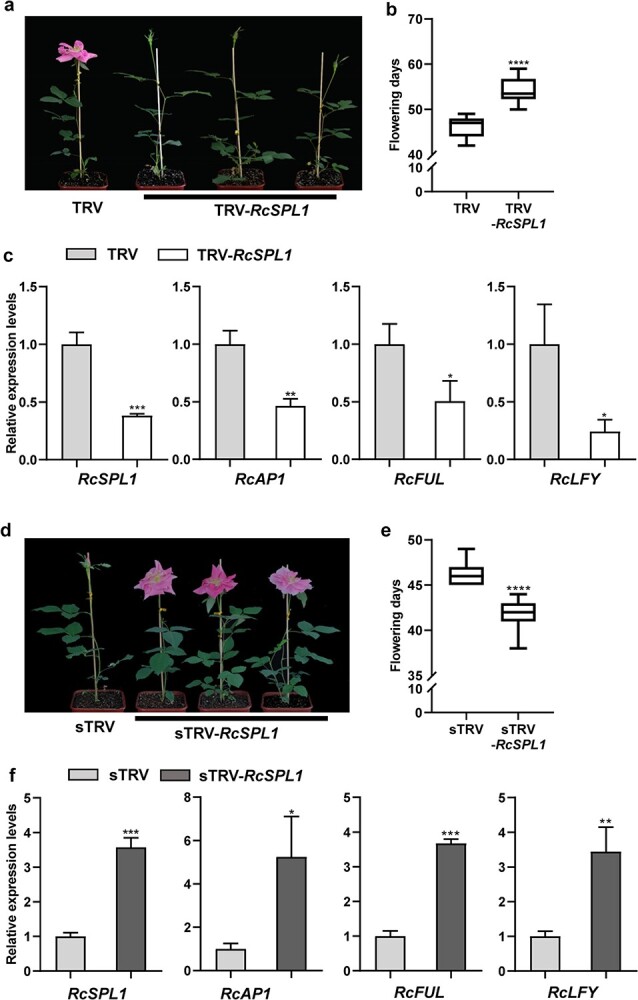
Changed flowering phenotypes displayed by the *RcSPL1-*silenced or -overexpression rose plants. **a**, **b** Comparison of flowering phenotype (**a**) and flowering days (**b**) of the empty vector control (TRV) with that of *RcSPL1*-silenced (TRV-*RcSPL1*) rose plants, respectively. The VIGS experiments were performed three times with different batches of rose plants, with similar results. For each experiment, at least 20 plants of TRV control or TRV-*RcSPL1* were used for phenotype observation (*n* ≥ 20). Representative results from one experiment are shown. **c** RT–qPCR analyses of *RcSPL1*, *RcAP1*, *RcFUL*, and *RcLFY* transcript levels in the leaves of TRV and TRV-*RcSPL1* rose plants. Mean values ± standard deviation are shown from three biological replicates (*n* = 3). *RcUBI2* was used as an internal control. **d**, **e** Flowering phenotype (**d**) and flowering days (**e**) of the empty vector control (sTRV) and *RcSPL1*-overexpressing (sTRV-*RcSPL1*) rose plants. The gene overexpression experiments were performed for three times with different batches of rose plants, with similar results. For each experiment, at least 20 plants of TRV control or TRV-*RcSPL1* plants were used for phenotype observation (*n* ≥ 20), and representative results from one experiment are shown. **f** RT–qPCR analyses of *RcSPL1*, *RcAP1*, *RcFUL*, and *RcLFY* transcription in the leaves of sTRV and sTRV-*RcSPL1* rose plants. Mean values ± standard deviation are shown from three biological replicates (*n* = 3). *RcUBI2* was used as the internal control. Asterisks represent statistically significant differences determined by Student’s *t-*test (^*^P < 0.05, ^**^P < 0.01, ^***^P < 0.001, ^****^P < 0.0001).

To clarify the effect of RcSPL1 on rose floral development, *RcSPL1-*silenced and -overexpressed *Rosa hybrida* ‘Samantha’ plants were generated using a TRV-mediated transient silencing and overexpressing system. The *RcSPL1* cDNA fragment (343 bp) was inserted into the pTRV2 virus-induced gene silencing (VIGS) vector, whereas the *RcSPL1* open reading frame (ORF) was cloned into the psTRV2 vector [43]. Changes in *RcSPL1* expression were preliminarily analyzed by RT–qPCR. Approximately 20 plants with downregulated or upregulated *RcSPL1* expression levels were used for the phenotypic analysis. Three plants with similar *RcSPL1* expression levels were selected for RT–qPCR assay (Supplementary Data Fig. S6). Compared with the corresponding control plants (TRV and sTRV), *RcSPL1* transcript levels were significantly lower in the *RcSPL1*-silenced (TRV-*RcSPL1*) rose plants, but substantially higher in the *RcSPL1*-overexpressed (sTRV-*RcSPL1*) rose plants (Fig. 4c and f). Consistent with the *RcSPL1* expression levels, the flowering of TRV-*RcSPL1* and sTRV-*RcSPL1* plants was significantly delayed and accelerated, respectively (Fig. 4a, b, d, e). More specifically, flowering was observed at 46.10 ± 2.25 days for the TRV control plants, which was ~8 days earlier than the flowering of the *RcSPL1-*silenced plants (54.05 ± 2.78 days) (Fig. 4b). Conversely, the flowering of sTRV-*RcSPL1* rose plants (41.85 ± 1.69 days) occurred significantly earlier (~5 days) than the flowering of the control plants (46.25 ± 1.40 days) (Fig. 4e). Moreover, to determine the influence of *RcSPL1* on vegetative phase transition of rose as in transgenic *Arabidopsis*, the expression levels of miR156 and *SPL1* were further detected during the development of ‘Mount Shasta’ (CF type) and *Rosa multiflora* (OF type) seedlings from the emergence of leaves to flowering by RT–qPCR. The results showed that the expression levels of rch-miR156 decreased, while *RcSPL1* increased (Supplementary Data Fig. S7). These results reflected the involvement of RcSPL1 in the control of flowering time and its possible association with the vegetative phase transition in rose.

### Altered *RcSPL1* expression affected the expression of flowering-related genes

To determine whether RcSPL1 affects the expression of the downstream genes, including *AP1*, *LFY*, and *FRUITFULL* (*FUL*), we analyzed gene expression levels in transgenic *A. thaliana* and rose plants. Compared with the control plants, the *AtAP1*, *AtFUL*, and *AtLFY* expression levels were significantly higher in the *RcSPL1*-OE lines (Fig. 3g–i). Similarly, the expression levels of their rose homologs (*RcAP1*, *RcFUL*, and *RcLFY*) increased in the *RcSPL1*-overexpressed plants (Fig. 3f), but decreased in the *RcSPL1-*silenced plants (Fig. 3c). In addition, similar to the trend in *RcSPL1* expression, the *RcAP1*, *RcFUL*, and *RcLFY* expression levels in ‘Old Blush’ and *R. chinensis* var. *spontanea* increased continuously during the floral development period (Supplementary Data Fig. S8). These results imply that RcSPL1 regulates rose floral development by activating the expression of the flowering-related genes *RcAP1*, *RcLFY*, and *RcFUL*.

### RcSPL1 interacted with RcTAF15b, a key regulator in the autonomous pathway

**Figure 5:**
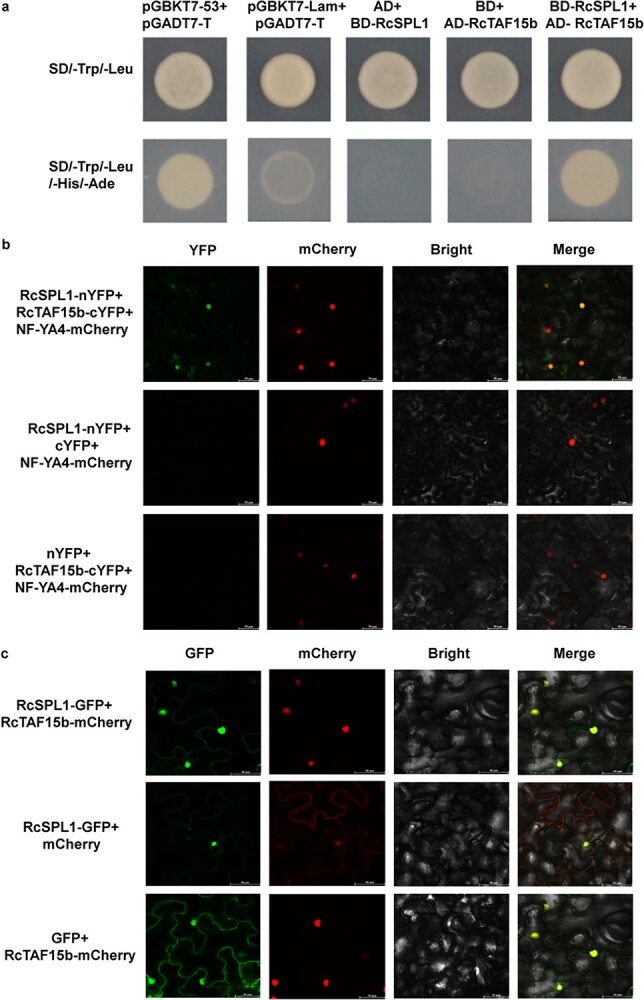
RcSPL1 interacts with RcTAF15b. **a** Interaction between RcSPL1 and RcTAF15b by Y2H. AD is empty vector of pGADT7 AD, BD is empty vector of pGBKT7 BD. Positive control was performed using pGBKT7-53 + pGADT7-T, while negative control was pGBKT7-Lam + pGADT7-T. Experiments were performed independently twice, with similar results. **b** Interaction of RcSPL1 and RcTAF15b in a BiFC assay. RcSPL1-nYFP was co-infiltrated with RcTAF15b-cYFP in *N. benthamiana* leaves. Infiltrated leaves were visualized by confocal microscopy 3 days after infiltration. Co-infiltrations of RcSPL1-nYFP with cYFP and of nYFP with RcTAF15b-cYFP were used as negative controls. pSuper:*NF-YA4*-mCherry was co-infiltrated as a nuclear marker. Scale bar, 50 μm. The experiment was performed independently three times, with similar results. Representative results from one experiment are shown. **c** RcSPL1 co-localization with RcTAF15b in tobacco leaves. RcSPL1-GFP and RcTAF15b-mCherry were transiently co-expressed in tobacco leaves. Co-localizations of RcSPL1-GFP with mCherry and of GFP with RcTAF15b-mCherry were used as negative controls. Scale bar, 50 μm. The experiment was performed independently three times, with similar results. Representative results from one experiment are shown.

To further examine the regulatory effects of RcSPL1 on flower development, we searched for proteins that can interact with RcSPL1. Preliminary screening of a rose cDNA library using a yeast two-hybrid (Y2H) system resulted in the detection of 23 proteins that may interact with RcSPL1. Among these proteins, four were identified as interacting proteins at least twice (Supplementary Data Table S4). Specifically, TAF15b was identified four times as a protein capable of interacting with RcSPL1 (Fig. 5a). The phylogenetic and conserved domain analyses showed that RcTAF15b is closely related to *A. thaliana* protein AtTAF15b and *F. vesca* protein FvTAF15b, which contained one Zn-finger-like structural motif and an RNA recognition motif (Supplementary Data Fig. S9a and b).

To verify the interaction between RcSPL1 and RcTAF15b, a bimolecular fluorescence complementation (BiFC) assay was conducted using *N. benthamiana*. A strong reconstituted yellow fluorescent protein (YFP) signal was observed in nuclei co-expressing RcSPL1-nYFP and RcTAF15b-cYFP (Fig. 5b). To determine whether these two proteins function in the same subcellular location, a co-localization assay was performed. Tobacco leaves were co-infiltrated with RcSPL1-GFP and RcTAF15b-mCherry. Subsequent examination indicated that RcSPL1 and RcTAF15b were both localized in the nucleus (Fig. 5c). These findings suggest that RcSPL1 controls flowering time possibly through its interaction with RcTAF15b, an important component of the autonomous pathway.

### Altered *RcTAF15b* expression influenced rose flowering

**Figure 6:**
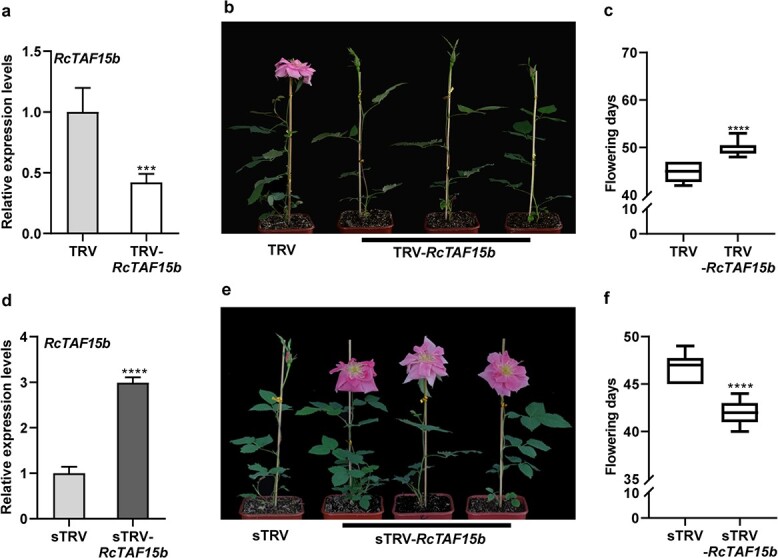
Effects of silencing or overexpressing of *RcTAF15b* on flowering phenotype. **a** RT–qPCR analyses of *RcTAF15b* transcript levels in the leaves of TRV and TRV-*RcTAF15b* plants. Mean values ± standard deviation are shown from three biological replicates (*n* = 3). *RcUBI2* was used as the internal control. **b**, **c** Flowering phenotype (**b**) and flowering days (**c**) of TRV control and *RcTAF15b*-silenced (TRV-*RcTAF15b*) plants. The VIGS experiments were performed three times, for different batches of rose plants, with similar results. For each experiment, at least 20 TRV control plants or TRV- *RcTAF15b* plants were used for phenotype observation (*n* ≥ 20). One representative result is shown. **d** RT–qPCR analyses of *RcTAF15b* transcript levels in leaves of sTRV and sTRV-*RcTAF15b* plants. Mean values ± standard deviation are shown from three biological replicates (*n* = 3). *RcUBI2* was used as the internal control. **e**, **f** Flowering phenotype (**e**) and flowering days (**f**) of sTRV control and *RcTAF15b*-overexpressed (sTRV-*RcTAF15b*) plants. The gene overexpression experiments were performed three times for different batches of rose plants, with similar results. At least 20 plants of TRV control or TRV- *RcTAF15b* were used for phenotype observation in each experiment (*n* ≥ 20). One representative result is shown. Asterisks represent statistically significant differences determined by Student’s *t*-test (^***^P < 0.001, ^****^P < 0.0001).

The observed interaction between RcTAF15b and RcSPL1 compelled us to assess whether RcTAF15b is involved in rose floral development. RT–qPCR analysis confirmed that the *RcTAF15b* transcript levels during the floral development period of ‘Old Blush’ and *R. chinensis* var. *spontanea* were consistent with the trends in *RcSPL1* transcript abundance (Supplementary Data Fig. S10), suggestive of a role for RcTAF15b during the flowering of rose plants. The analysis of the *RcTAF15b*-silenced (TRV-*RcTAF15b*) and -overexpressed (sTRV-*RcTAF15b*) rose plants revealed that the *RcTAF15b* transcript level was much lower in the *RcTAF15b*-silenced plants than in the TRV control plants (Fig. 6a, Supplementary Data Fig. S10), but it was significantly higher in the sTRV-*RcTAF15b* plants than in the sTRV control plants (Fig. 6d, Supplementary Data Fig. S11). As expected, compared with the control plants, which flowered at 44.70 ± 2.06 days, the *RcTAF15b*-silenced plants flowered ~5 days later (49.70 ± 1.64 days) (Fig 6b and c). The *RcTAF15b*-overexpressed rose plants flowered earlier (41.85 ± 1.27 days) than the sTRV control plants (46.65 ± 1.42 days) (Fig 6e and f). These results reflected the importance of RcTAF15b for the flowering of rose plants.

## Discussion

Rose, which is one of the most popular ornamental flower species, can be divided into three types: recurrent or continuous flowering (CF), occasional re-blooming (OR), and once-flowering (OF). Because of its unique and diverse floral habits, rose is a good model for investigating the molecular mechanism underlying the flowering of perennial plants. Therefore, identifying the key flowering regulators in rose may help researchers and breeders develop superior perennial plant varieties that can flower continuously. In this study, we determined that the interaction between RcSPL1 and RcTAF15b facilitates the co-regulation of rose floral development.

There are 15 *SPL* genes in rose (Supplementary Data Table S1), but in this study only *RcSPL1* transcript levels were continuously upregulated during the rose flower development period (Fig. 1a). Overexpressing *RcSPL1* in *A. thaliana* promoted the emergence of abaxial trichomes and the serration of the leaf margins (Fig. 3b), suggesting that RcSPL1 might play a similar function by accelerating the juvenile-to-adult transition in rose. In *A. thaliana*, decreases in the miR156 level or the overexpression of its targets (*SPL* genes) can accelerate vegetative and reproductive phase transitions [41, 44]. Similarly, the upregulated expression of *SPL* genes promotes the production of deltoid leaves (a marker of the vegetative phase transition) in poplar (*Populus* × *canadensis*) [45]. In addition, previous studies showed that the age pathway is mainly controlled by miR156 and its target transcription factors, *SPL*s; the expression of miR156 gradually decreases as *SPL* gene expression increases during the transition to flowering [15, 16]. Moreover, rch-miR156 and *RcSPL1* exhibited opposite expression patterns during the development of rose seedlings (Supplementary Data Fig. S7). Therefore, rch-miR156/*RcSPL1* may be a master regulator of the age pathway in rose. Notably, the rch-miR156 transcript levels in ‘Old Blush’ and *R. chinensis* var. *spontanea* gradually decreased during the flower development period, which was in contrast to the changes in *RcSPL1* expression (Fig. 1a, Supplementary Data Fig. S12), indicating that rch-miR156 may also participate in rose floral development. Furthermore, overexpressing *RcSPL1* in *A. thaliana* and rose plants led to a marked decrease in flowering time (Figs 3d and e and 4d and e). Conversely, the silencing of *RcSPL1* resulted in a late-flowering phenotype (Fig. 4a and b). Accordingly, RcSPL1 is a key factor influencing the timing of rose flowering. In addition to regulating the vegetative phase transition and flowering, SPL family members also have multiple functions affecting diverse processes related to growth and development, such as plant architecture and fruit ripening in various plant species [27, 46]. These findings indicate that different *SPL* genes may encode functionally diverse proteins. The 14 other *RcSPL* genes will need to be functionally validated in future studies.

The *AP1*, *FUL*, and *LFY* genes have critical effects on flowering induction, floral organ development, inflorescence, and floral meristem identity [47, 48]. In our study, *RcAP1*, *RcFUL*, and *RcLFY* expression was significantly activated by RcSPL1. The transcripts of these genes were more highly accumulated in the transgenic *A. thaliana* lines and the *RcSPL1*-overexpressed rose plants than in their corresponding control plants, but the transcripts were accumulated at lower levels in the *RcSPL1*-silenced rose plants (Figs 3g–i and 4c and f). Therefore, altering the expression of *RcSPL1* disrupted the expression of *RcAP1*, *RcFUL*, and *RcLFY*. Previous research demonstrated that SPL proteins regulate the transcript accumulation of downstream genes through the binding of the SBP domain to the GTAC core motif [49]. Indeed, several GTAC-boxes exist in the *RcAP1*, *RcFUL*, and *RcLFY* promoters according to Plantpan3.0 (http://plantpan.itps.ncku.edu.tw/index.html), suggesting that RcSPL1 may bind directly to the promoters to regulate the flowering time in rose.

The current reports suggested that *SPL* can link to multiple flowering signals derived from photoperiodic, GA, and vernalization pathways to control flowering. In *Arabidopsis*, SOC1 directly regulates the expression of *SPL3*, *SPL4*, and *SPL5* by binding to their promoters in response to photoperiod signals [50]. In the GA flowering pathway, DELLA proteins are recruited to the *AP1* locus by SPL9, which induces the expression of *AP1* and promotes the transition of lateral primordia to flowers [51]. In *Arabis alpina* and *Cardamine flexuosa*, age regulates the timing of sensitivity in response to vernalization, which is associated with the miR156-SPL-miR172 cascade [15, 52, 53]. In the current study, we revealed the interaction between RcSPL1 and RcTAF15b and determined that the resulting complex is localized in the nucleus (Fig. 5). Generally, SPLs, which are the key regulators of the age pathway, and TAF15b, which is a typical autonomous pathway protein, are involved in the regulation of reproductive processes [16, 33]. Our results on the interaction between RcSPL1 and RcTAF15b may imply crosstalk of the age and autonomous pathways in flowering time regulation. Transiently silencing or overexpressing *RcTAF15b* in rose via TRV resulted in late or early flowering, respectively (Fig. 6), indicating RcTAF15b contributes to rose floral transition. In *A. thaliana*, TAF15b promotes flowering by repressing the transcription of *FLOWERING LOCUS C* (*FLC*), which is a flowering repressor [33]. However, both ‘Old Blush’ and *R. chinensis* var. *spontanea* lack the *FLC* gene [1, 54]. In rose, both RcSPL1 and RcTAF15b promote early flowering. Thus, we speculate that RcTAF15b acts synergistically with RcSPL1 to co-regulate the expression of the floral meristem identity genes *RcAP1*, *RcFUL*, and *RcLFY*. Further studies will be required to clarify whether RcTAF15b directly regulates flowering-related genes in rose.

### Conclusions

The study provides new insights into the mechanisms regulating flowering time. Specifically, *RcSPL1*, a member of the *SPL* family, exhibited continuous increased expression during the rose flowering process and controls the expression of the floral meristem identity genes, *RcAP1*, *RcFUL*, and *RcLFY*. Additionally, its upstream regulator of rch-miR156 and the interacting partner RcTAF15b were also identified. These results help to understand the mechanism underlying rose flowering control via RcSPL1 and RcTAF15b synergistically regulating flowering time, with potential implications for the breeding and cultivation of rose or other perennial plants.

## Materials and methods

### Plant materials and growth conditions

*R. chinensis* ‘Old Blush’ and *R. chinensis* var. *spontanea* buds were obtained from the Daxing Rose Garden (Beijing, China) and mountains in Mianyang (Sichuan, China), respectively. Multiple buds (~0.2 g) at each stage were pooled as one biological replicate. In addition, *Rosa hybrida* ‘Samantha’ plants (CF type) were propagated via an *in vitro* culture system. Rose stems (explants) were cultured on propagation medium [Murashige and Skoog (MS), 1.0 mg l^−1^ 6-benzylaminopurine (6-BA), 0.01 mg l^−1^ 1-naphthaleneacetic acid (NAA), 30 g l^−1^ sucrose, 3 mg l^−1^ gibberellic acid (GA_3_), and 7.5 g l^−1^ agar, pH 5.8] for ~1 month, after which they were transferred to rooting medium [MS, 0.1 mg l^−1^ NAA, 30 g l^−1^ sucrose, and 7.5 g l^−1^ agar, pH 5.8] for ~1 month. The plants were grown at 22 ± 1°C with a 16-hour light/8-hour dark photoperiod and 50% relative humidity.

WT and transgenic *A. thaliana* plants and *N. benthamiana* plants were grown at 25°C with a 16-hour light/8-hour dark photoperiod and 60% relative humidity.

### Rose morphological examination

‘Old Blush’ samples at stages 1–3 were fixed in FAA solution (formaldehyde:acetic acid:50% ethanol = 5:5:90 v/v) and then dehydrated in a graded ethanol series before being embedded in paraffin. Sections (0.5–0.8 μm) prepared using a microtome (Leica RM2265) were deparaffinized using xylene and rehydrated in a graded ethanol series. Finally, the sections were stained with toluidine blue and examined using an inverted microscope (Zeiss Observer Z1).

*R. chinensis* var. *spontanea* (stages 4–6) were carefully dissected using a binocular stereomicroscope and then fixed in 2.5% glutaraldehyde (v/v) for >2 h under vacuum conditions. The samples were rinsed with 0.1 ml phosphate-buffered saline (PBS), followed by a post-fixation treatment with 1% osmium tetroxide. The samples were rinsed again with 0.1 ml PBS and then dehydrated using a graded alcohol series and a critical point dryer (Leica EM CPD). Finally, samples were coated with gold (EIKO IB-3) and examined using a scanning electron microscope (Hitachi S-3400 N).

### RNA extraction and RT–qPCR

Total RNA was isolated using the FastPure Plant Total RNA Isolation Kit (Vazyme, Nanjing, China) and then reverse-transcribed to cDNA using the HiScript III RT SuperMix for qPCR (+ gDNA wiper) kit (Vazyme). First-strand miRNA was reverse-transcribed using step-loop primers and the miRNA 1st Strand cDNA Synthesis Kit (Accurate Biology, China). The RT–qPCR analysis was performed using the ChamQ Universal SYBR qPCR Master Mix (Vazyme), with *AtActin* and *RcUBI2* used as the reference genes for *A. thaliana* and *R. chinensis*, respectively. Additionally, 5.8S rRNA and U6 snRNA were used to normalize the abundance of miRNA in *R. chinensis* and *N. benthamiana*, respectively. All primers used in this study are listed in Supplementary Data Table S5. The significance of any differences in the data was evaluated using SPSS software (version 22.0 for Windows; Chicago, IL, USA) and GraphPad Prism (version 9.0). Student’s *t*-test, ANOVA, or Duncan’s multiple range test was used to analyze the experimental data.

### Sequence and phylogenetic analysis

The BLAST (https://blast.ncbi.nlm.nih.gov/Blast.cgi) and TAIR (https://www.arabidopsis.org/) databases were screened for homologous DNA and protein sequences. The amino acid sequences were aligned and phylogenetic analysis was performed using ClustalW (https://www.genome.jp/tools-bin/clustalw) and MEGA X [55], respectively. Phylogenetic trees were constructed according to the neighbor-joining method with 1000 bootstrap replicates. Sequence logos were created using the WebLogo platform (http://weblogo.threeplusone.com/).

### Subcellular localization

The *RcSPL1* coding sequence (CDS) was fused to the sequence encoding GFP and inserted into the pCAMBIA1300 vector containing the Super promoter to construct the pSuper:*RcSPL1*-GFP plasmid. pSuper:*NF-YA4*-mCherry was applied as a nuclear marker [39]. The recombinant plasmids were introduced into *Agrobacterium tumefaciens* strain GV3101 cells by electroporation. *A. tumefaciens* harboring different plasmids was resuspended in MMA infiltration buffer (10 mM MgCl_2_, 10 mM MES, and 100 μM acetosyringone, pH 5.6; OD_600_ = 0.8). *N. benthamiana* leaves were co-infiltrated with the *A. tumefaciens* suspensions. After 3 days, fluorescent signals were detected using a laser confocal microscope (Leica TCS SP8). The primers used in this study are listed in Supplementary Data Table S5.

### Prediction of *RcSPL1* targeted by rch-miR156

The previously reported rch-miR156 sequence was obtained [56], whereas the rch-miR156 precursor sequence in rose was downloaded from the ‘Old Blush’ pre_miRNA database (https://lipm-browsers.toulouse.inra.fr/pub/RchiOBHm-V2/). The *RcSPL1* gene potentially targeted by miR156 was identified from the 3′UTR of *RcSPL1* for sequences complementary to rch-miR156 using the default parameters of the psRNATarget server (https://www.zhaolab.org/psRNATarget/). The predicted cleavage site in *RcSPL1* and the primary of rch-miR156 (pri-rch-miR156) transcript were amplified separately by PCR and cloned into the pCAMBIA1300 vector with GFP (130-GFP vector) or without GFP (pCAMBIA1300 vector) to construct the p35S:*RcSPL1-sensor*-GFP and p35S:*pri-rch-miR156* recombinant plasmids, respectively. The plasmids were introduced into *A. tumefaciens* GV3101 cells by electroporation. Cultures of *A. tumefaciens* cells containing p35S:*RcSPL1-sensor*-GFP (RcSPL1-sensor-GFP), pCAMBIA1300, or 130-GFP (OD_600_ = 0.5), and cultures of *A. tumefaciens* cells containing p35S:*pri-rch-miR156* (pri-rch-miR156) (OD_600_ = 0.2, 0.5, and 0.8) were prepared. Cultures of RcSPL1-sensor-GFP with pri-rch-miR156 or pCAMBIA1300 in a 1:1 (v/v) ratio were used for the infiltration of *N. benthamiana* leaves. After 2–3 days, the percentage of cells with GFP signal in the same-size microscopic field was determined. Specifically, GFP fluorescence was detected using FUSION FX EDGE SPECTRA and a laser confocal microscope (Leica STED). The abundance of rch-miR156 in *N. benthamiana* leaves was quantified on the basis of an RT–qPCR analysis. The *RcSPL1* ORF with or without the 3′UTR was amplified by PCR and inserted into 130-DAS-His vector at the 3′ end of the 6× His-tag encoding sequence to construct the p35S:His-*RcSPL1-3′UTR* and p35S:His-*RcSPL* recombinant plasmids, respectively. All of the recombinant constructs were expressed in *N. benthamiana* leaves. After 3 days, the His-fused protein was detected. The experiments were repeated three times.

### *Arabidopsis thaliana* transformation

The *RcSPL1* CDS was amplified by PCR and inserted into the SacI and XbaI sites of the pBI121 vector to construct the p35S:*RcSPL1* recombinant plasmid, which was subsequently introduced into *A. tumefaciens* cells for the transformation of *A. thaliana* via the floral dip method [57]. Independent transgenic *A. thaliana* lines were screened on MS basal medium supplemented with 40 mg l^−1^ kanamycin. The putative transformants were confirmed via a PCR amplification. The *T*_2_ generation plants were used for further analysis. To detect target gene expression, the rosette leaves of 3-week-old *A. thaliana* plants were collected and immediately frozen in liquid nitrogen.

### Virus-induced gene silencing and overexpression in rose

To silence *RcSPL1* and *RcTAF15b*, gene-specific fragments of *RcSPL1* (343 bp) and *RcTAF15b* (305 bp) were cloned into separate pTRV2 vectors to obtain the pTRV2-*RcSPL1* and pTRV2-*RcTAF15b* recombinant plasmids. The *RcSPL1* and *RcTAF15b* genes were silenced in rose plantlets as previously described [39]. Briefly, cultures of *A. tumefaciens* cells transformed with pTRV1, pTRV2-*RcSPL1*, pTRV2-*RcTAF15b*, or pTRV2 (negative control) were prepared (OD_600_ = 1.2), after which the cells were resuspended in MMA buffer supplemented with 0.001% (v/v) Silwet-L77 (OD_600_ = 1.0). Finally, solutions comprising cells containing pTRV1 and cells containing pTRV2-*RcSPL1*, pTRV2-*RcTAF15b*, or pTRV2 (equal ratio; v/v) were prepared. Before infiltrating plants, the mixtures were kept in darkness at room temperature for 3–5 hours. Whole plants were submerged in infiltration buffer and exposed to vacuum conditions (−20 kPa) twice, each for 1 minute, and then the plants were rinsed with distilled water and transferred to pots. After 3–4 weeks, new leaves were collected from the treated plants and immediately frozen in liquid nitrogen. The transcription of the target genes (*RcSPL1* or *RcTAF15b*) in each plant was detected by performing an RT–qPCR analysis. Approximately 20 plants in which the expression levels of the target genes were downregulated were selected for phenotypic examination.

Rose plants overexpressing the target genes were created using psTRV1 and psTRV2 plasmids [43]. *RcSPL1* and *RcTAF15b* ORF fragments containing a KpnI restriction site were inserted into psTRV2 to obtain psTRV:*RcSPL1* and psTRV:*RcTAF15b* recombinant plasmids, respectively. The *RcSPL1* and *RcTAF15b* genes were transiently overexpressed in rose plantlets via the above-mentioned operation of the VIGS experiment. Plants infiltrated with the empty psTRV vectors (psTRV1 + psTRV2) were used as the sTRV control plants. After 3–4 weeks, new leaves were collected from each treated plant and immediately frozen in liquid nitrogen. RT–qPCR analysis was completed to confirm that the target genes were more highly transcribed in the gene-overexpressing rose plants than in the control plants. Finally, ~20 plants in which the target gene expression levels were upregulated were selected for phenotypic examination. Both TRV-mediated gene silencing and overexpression experiments were performed three times using different batches of rose plants, with each batch comprising at least 70 plantlets.

### Yeast two-hybrid assay

A Y2H assay was performed to screen a rose floral bud cDNA library for RcSPL1-interacting proteins [58]. The *RcSPL1* ORF was cloned into pGBKT7 BD to construct the bait vector, whereas the *RcTAF15b* ORF was inserted into pGADT7 AD to generate the prey vector. The Y2H assay was completed using the Matchmaker™ Gold Yeast Two-Hybrid system. As positive and negative controls, Y2HGold yeast cells were co-transformed with pGADT7-T and pGBKT7-53 or pGBKT7-Lam. The transformants were grown on SD/−Trp/−Leu and SD/−Trp/−Leu/−His/−Ade media for 3 days at 30°C.

### Bimolecular fluorescence complementation assay

For the BiFC assay, the *RcSPL1* ORF was inserted into pSYNE-35S, which contains the sequence encoding the N terminus of YFP (nYFP), while the *RcTAF15b* ORF was inserted into pSPYCE-35S, which contains the sequence encoding the C terminus of YFP (cYFP). pSuper:*NF-YA4*-mCherry was used as a nuclear marker [39]. A mixture comprising *A. tumefaciens* cells harboring *RcSPL1*-nYFP and *RcTAF15b*-cYFP was used for the co-infiltration of *N. benthamiana* leaves. The following combinations were used as negative controls: RcSPL1-nYFP + cYFP and nYFP + RcTAF15b-cYFP. After 3 days, the leaves were examined for YFP and mCherry signals.

### Co-localization assay

For the co-localization assay, the pSuper:*RcSPL1*-GFP recombinant plasmid was constructed. The *RcTAF15b* CDS fused to the mCherry sequence was inserted into the pCAMBIA1300 vector to produce the p35S:*RcTAF15b*-mCherry recombinant plasmid. *Nicotiana benthamiana* leaves were co-infiltrated with pSuper:*RcSPL1*-GFP and p35S:*RcTAF15b*-mCherry. The following combinations were used as negative controls: pSuper:*RcSPL1-*GFP + mCherry and pSuper:GFP + p35S:*RcTAF15b*-mCherry. After 3 days, the leaves were examined for red and green fluorescence.

## Acknowledgements

This work was funded by Guest Investigator Grant of the State Key Laboratory of Plant Genomics, Institute of Microbiology, Chinese Academy of Science (SKLPG2016A-29). We thank Professor Nan Ma (China Agricultural University) for providing the TRV1, TRV2, pSuper1300-GFP, and pSuper:*NF-YA4*-mCherry plasmids, as well as cDNA library of Y2H. We also thank Professor Jian Ye (Institute of Microbiology, Chinese Academy of Sciences) for kindly providing the psTRV1 and psTRV2 vectors. We thank Liwen Bianji (Edanz) (www.liwenbianji.cn) for editing the English text of a draft of this manuscript.

## Author contributions

R.Y. designed and performed the experiments, analyzed the data, and wrote the manuscript. Z.Y.X. performed gene silencing and protein interaction assays; and X.H.Z. performed the overexpression assays; P.P.F. performed the RT–qPCR analyses and morphology observations; Z.Y.H. revised the manuscript; R.X.F. and Y.M.Z. guided the research and revised the manuscript; and Q.L.L. designed the experiments, conceived the project, and revised the manuscript. All authors read and approved the manuscript.

## Data availability

The sequencing data that support the findings of this study are available in the genome database of ‘Old Blush’ (https://lipm-browsers.toulouse.inra.fr/pub/RchiOBHm-V2/) and genome sequence archive (https://ngdc.cncb.ac.cn/search/?dbId=gwh&q=GWHAAAF00000000&page=1).

## Conflict of interest

The authors declare no competing interest.

## Supplementary data

Supplementary data is available at *Horticulture Research* online.

## Supplementary Material

Web_Material_uhad083
