# Supplementary material for: RcSPL1–RcTAF15b regulates the flowering time of rose (Rosa chinensis)
Source: Hortic Res. 2023 Apr 25;10(6):uhad083. doi: 10.1093/hr/uhad083 (PMC10266950; doi:10.1093/hr/uhad083)
Supplement: Web_Material_uhad083 [file web_material_uhad083.docx]

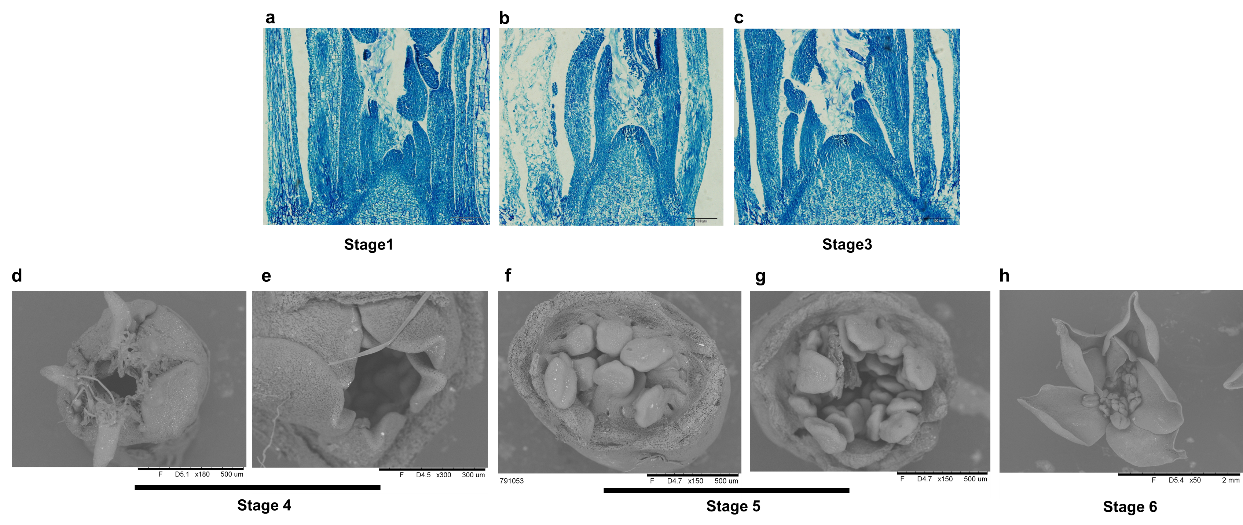


Supplementary Fig. 1 Rose flower development stages. (a-c) Morphology observation of floral transition in ‘Old blush’ with paraffin section. (a) Stage 1, the shoot apical meristem (SAM) is cone-shaped and narrow, named vegetative stage. (b) Stage 2, the meristem is flat-disk shape and enlarges laterally, which represents the vegetative-to-reproductive transition, named pre-floral stage. (c) Stage 3, the meristem becomes flatter, larger and doming structure, named floral stage. (d-h) Morphology observation of floral organ development in *R. chinensis* var. *spontanea* with scanning electron microscopy. (d) and (e) Stage 4, the sepal and petal primordia were visible. (f) and (g) Stage 5, the stamens and carpel primordia initiated and developed. (h) All floral organs are apparent. Due to the limited samples, only partial stages were preliminary assayed in ‘Old blush’ and *R. chinensis* var. *spontanea*, respectively, in that, the first three stages (stage 1-3) in ‘Old blush’ and the final three stages (stage 4-6) in *R. chinensis* var. *spontanea*. At least 20 buds of each stage were used for determining the stages of rose floral development.


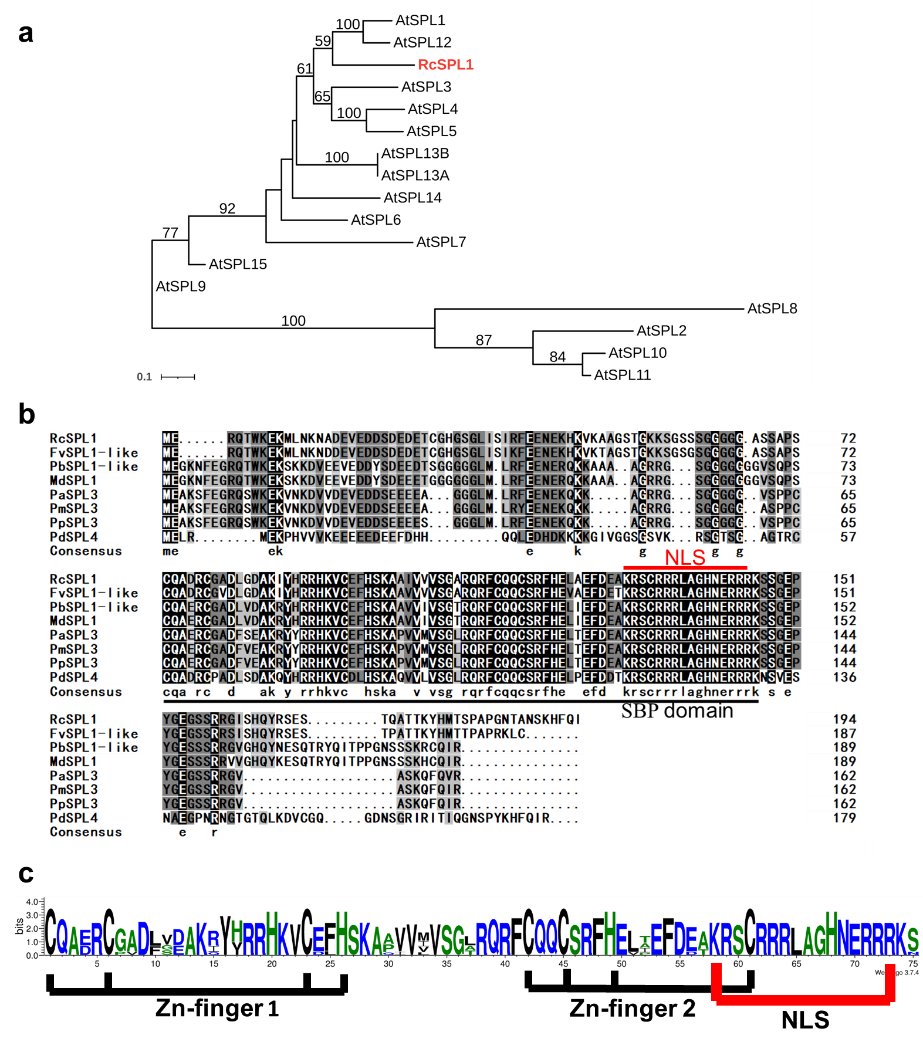


Supplementary Fig. 2 Comparison of the deduced amino acid sequence of RcSPL1 with SPL protein sequences from other plant species. (a) Phylogenetic analysis of RcSPL1 and SPLs from Arabidopsis. The phylogenetic tree was constructed using MEGA X with neighbor-joining algorithm. Bootstrap values indicate the divergence of each branch, and the scale bare indicates the branch length. AtSPL1 (AT2G47070), AtSPL2 (AT5G43270), AtSPL3 (AT2G33810), AtSPL4 (AT1G53160), AtSPL5 (AT3G15270), AtSPL6 (AT1G69170), AtSPL7 (AT5G18830), AtSPL8 (AT1G02065), AtSPL9 (AT2G42200), AtSPL10 (AT1G27370), AtSPL11 (AT1G27360), AtSPL12 (AT3G60030), AtSPL13A (AT5G50570), AtSPL13B (AT5G50670), AtSPL14 (AT1G20980), AtSPL15 (AT3G57920). (b) Multiple sequence alignment of RcSPL1 with SPL proteins from other plants species. The predicted SBP domain and NLS fragment are indicated by black and red solid lines, respectively. NLS, nuclear localization signal. FvSPL1-like (*Fragaria vesca* subsp. *Vesca*, XP_011458918.1), MdSPL1 (*Malus domestica*, XP_008386198.2), PaSPL3 (*Prunus avium*, XP_021821700.1), PbSPL1-like (*Pyrus* x *bretschneideri,* XP_009340759.1), PdSPL4 (*Prunus dulcis*, XP_034208663.1), PmSPL3 (*Prunus mume*, XP_008226482.1), PpSPL3 (*Prunus persica*, XP_007213489.1). (c) Sequence logo of the SBP domain of RcSPL1. Two conserved zinc (Zn)-finger structures and the NLS are indicated.


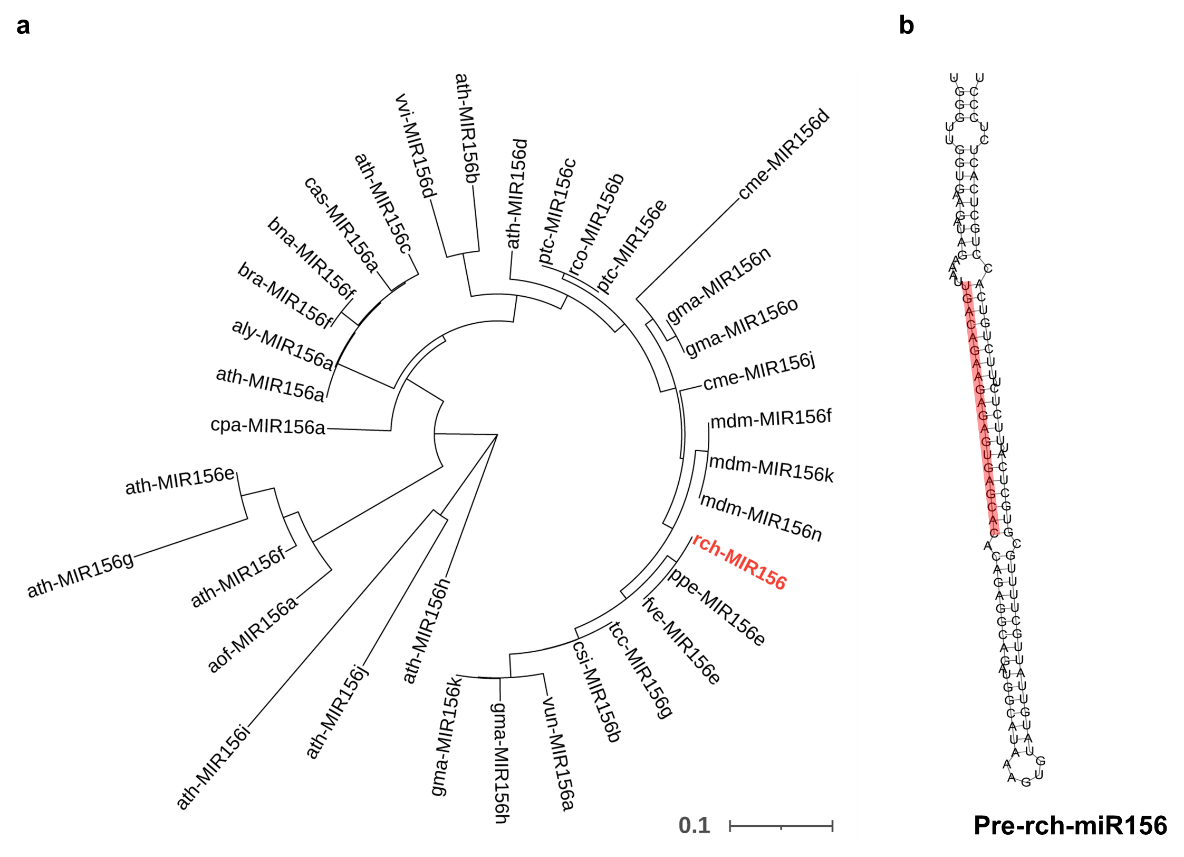


Supplementary Fig. 3 Characterization of rch-MIR156. (a) Phylogenetic tree of rch-MIR156 and the MIR156 from other plants. The precursor sequences of *MIR156* genes were retrieved from miRbase (http://www.mirbase.org/). The phylogenetic tree was constructed using MEGA X with neighbor-joining algorithm. The scale bare indicates the branch length. aly-MIR156a (*Arabidopsis lyrata,* MI0014502), aof-MIR156a (*Asparagus officinalis*, MI0040559), ath-MIR156a (*A. thaliana*, MI0000178), ath-MIR156b (*A. thaliana*, MI0000179), ath-MIR156c (*A. thaliana*, MI0000180), ath-MIR156d (*A. thaliana*, MI0000181), ath-MIR156e (*A. thaliana*, MI0000182), ath-MIR156f (*A. thaliana*, MI0000183), ath-MIR156g (*A. thaliana*, MI0001082), ath-MIR156h (*A. thaliana*, MI0001083), ath-MIR156i (*A. thaliana*, MI0019232), ath-MIR156j (*A. thaliana*, MI0019234), bna-MIR156f (*Brassica napus*, MI0020262), bra-MIR156f (*Brassica rapa*, MI0030552), cas-MIR156a (*Camelina sativa*, MI0037162), cme-MIR156d (*Cucumis melo*, MI0023215), cme-MIR156j (*Cucumis melo*, MI0023290), cpa-MIR156a (*Carica papaya*, MI0026328), csi-MIR156b (*Citrus sinensis*, MI0039615), fve-MIR156e (*F. vesca*, MI0036353), gma-MIR156h (*G. max*, MI0017819), gma-MIR156k (*Glycine max*, MI0018652), gma-MIR156n (*G. max*, MI0018655), gma-MIR156o (*G. max*, MI0018656), mdm-MIR156f (*Malus domestica*, MI0022976), mdm-MIR156k (*M. domestica*, MI0022981), mdm-MIR156n (*M. domestica*, MI0022984), ppe-MIR156e (*Prunus persica*, MI0026060), ptc-MIR156c (*Populus trichocarpa*, MI0002186), ptc-MIR156e (*P. trichocarpa*, MI0002188), rco-MIR156b (*Ricinus communis*, MI0013371), tcc-MIR156g (*Theobroma cacao*, MI0017459), vun-MIR156a (*Vigna unguiculata*, MI0019565), vvi-MIR156d (*Vitis vinifera*, MI0006488). (b) The precursor structure of rch-miR156 was predicted by RNAfold. miRNA sequences are highlighted in red.


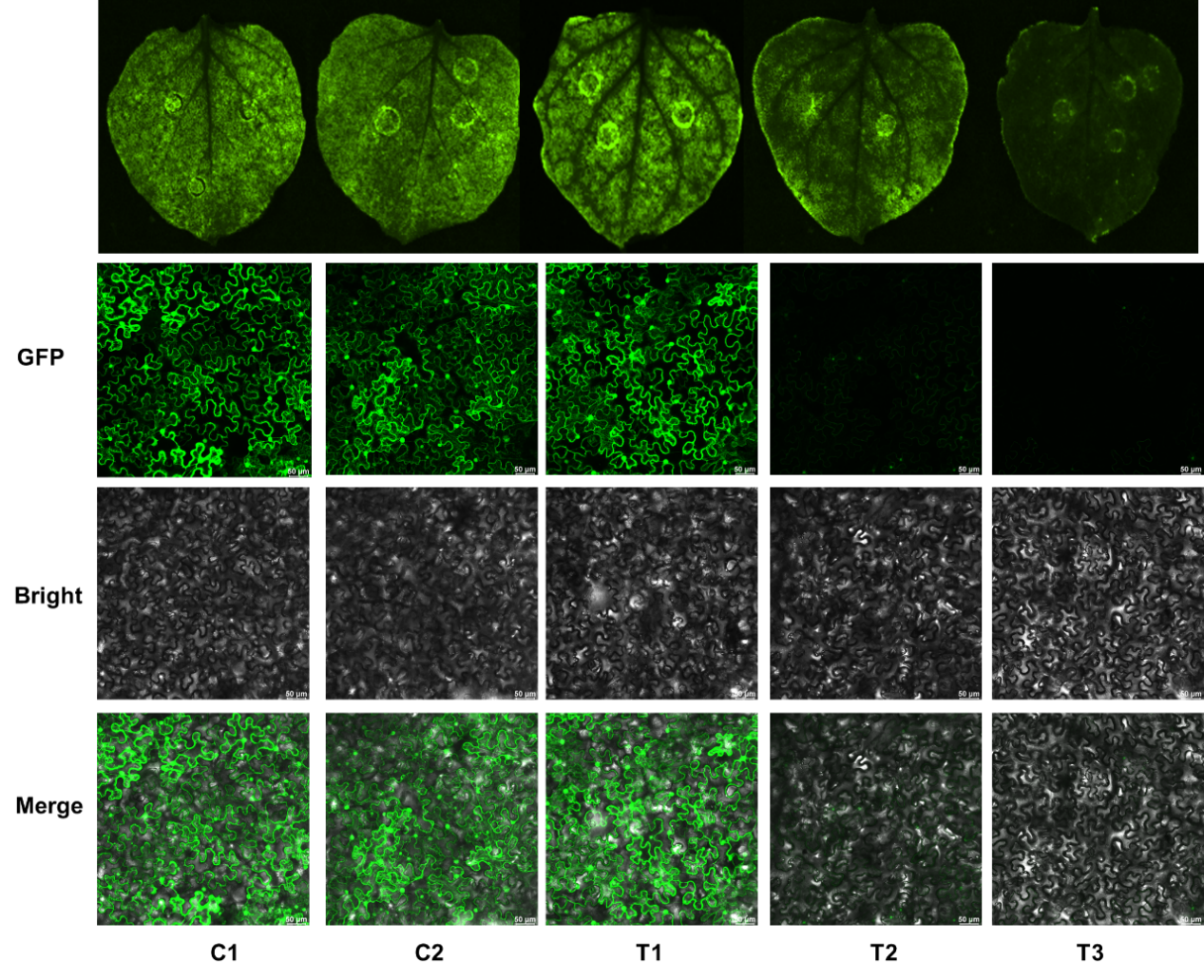


Supplementary Fig. 4 The GFP signal fluorescence in *N. benthamiana*. After infiltration, the GFP fluorescence was observed by FUSION FX. EDGE SPECTRA (upper) and laser confocal microscopy (Leica STED) (b). T1-T3: p35S:*RcSPL1-sensor*-GFP (RcSPL1-sensor-GFP, OD_600_ = 0.5) combined with varied concentrations of p35S:*pri-rch-miR156* (pri-rch-miR156, OD_600_ = 0.2, 0.5, or 0.8) constructs transiently co-expressed in the leaves of *N. benthamiana*. The pCAMBIA1300 + RcSPL1-sensor-GFP (C1) and pri-rch-miR156 + 130-GFP (C2) were acted as controls, and *A. tumefaciens* suspensions used in C1 and C2 with OD_600_ = 0.5. The experiments were performed independently 3 times, with similar results. The representative results are shown. Scale bar, 50 μm.


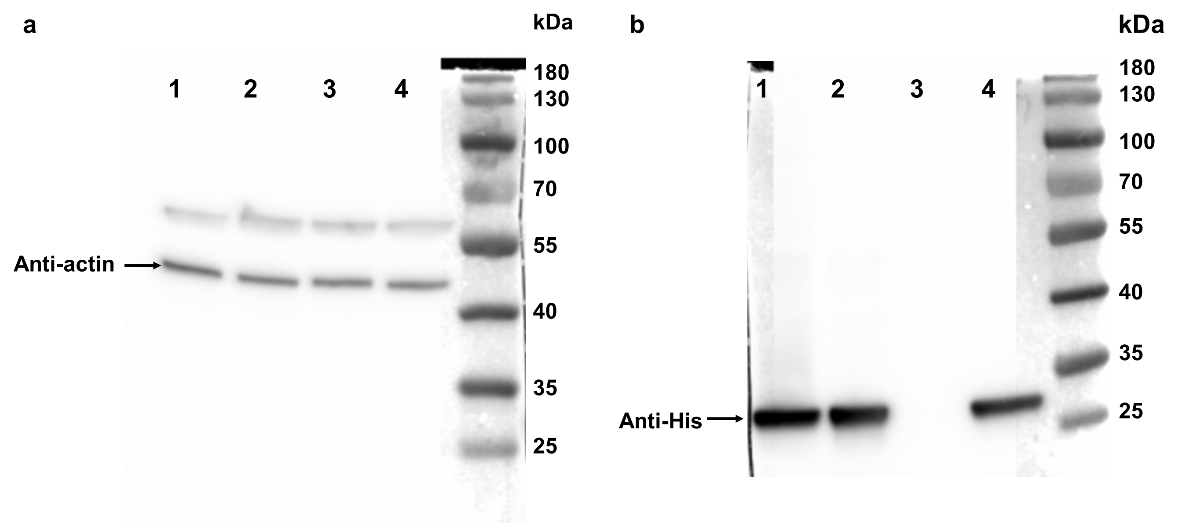


# Supplementary Fig. 5 p35S: His-*RcSPL1-3′UTR* (His-RcSPL1-3′UTR) and pri-rch-miR156 constructs transiently co-expresses in the leaves of *N. benthamiana*. (a and b) The proteins were analyzed by Western blotting (WB) using anti-actin (a) and anti-His antibodies (b), respectively. The order of proteins (1-4) was pCAMBIA1300 + His-RcSPL1, pri-rch-miR156 + His-RcSPL1, pri-rch-miR156 + His-RcSPL1-3′UTR, and pCAMBIA1300 + His-RcSPL1-3′UTR. The protein ladder (Thermo 26616) was used in the experiments. The experiments were performed independently 3 times, with similar results. The representative results from one experiment are shown.


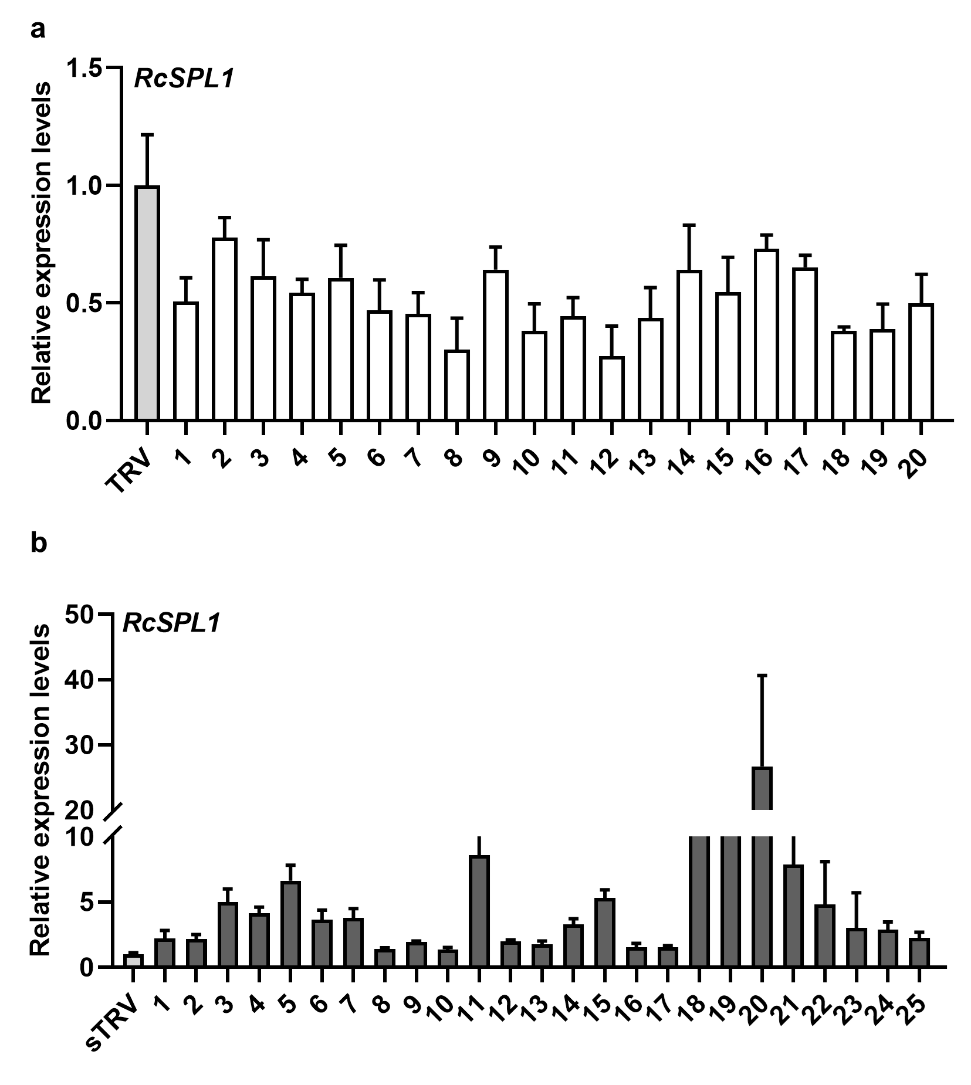


# Supplementary Fig. 6 Transcript levels of *RcSPL1* in leaves of silenced or overexpressed rose plants. (a) RT-qPCR analysis of *RcSPL1* expression in TRV control and *RcSPL1*-silenced (TRV-*RcSPL1*, No. 1-20) rose plants. *RcUBI2* was used as an internal control. Mean values ± SD are shown from three technical replicates. About 70 plantlets were performed in VIGS experiment, and about 20 plants with downregulated expression levels of *RcSPL1* were obtained and used for phenotype observation. Three plants of No.10, 18 and 19 were selected to further RT-qPCR. (b) RT-qPCR analysis of *RcSPL1* expression in sTRV control and *RcSPL1*-overexpressed (sTRV-*RcSPL1*, No. 1-25) rose plants. *RcUBI2* was used as the internal control. Mean values ± SD are shown from three technical replicates. About 70 plantlets were performed in gene overexpression experiment, and about 20 plants with upregulated expression levels of *RcSPL1* were obtained and used for phenotype observation. The No. 6, 7 and 14 plants were selected to further RT-qPCR. VIGS and gene overexpression experiments were performed three times independently, with similar results obtained.


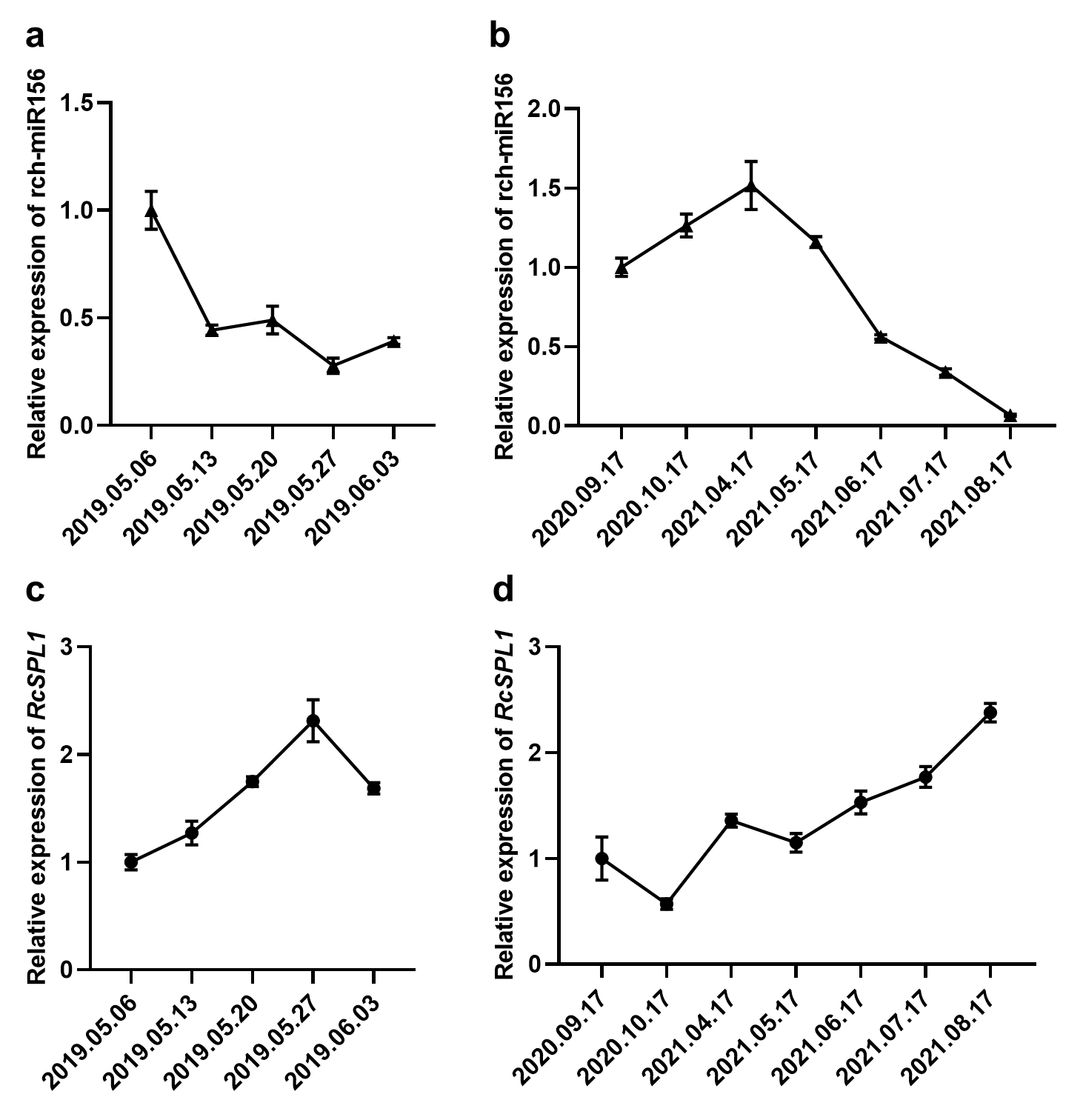


# Supplementary Fig. 7 RT-qPCR analyses of miR156 and *SPL1* transcript levels in leaves of differently aged *R. hybrid* ‘Mount Shasta’ and *Rosa multiflora* seedlings. (a and b) miR156 (a) and *SPL1* (b) transcript levels in leaves of differently aged *R. hybrid* ‘Mount Shasta’ seedlings. (c and d) miR156(c) and *SPL1* (d) transcript levels in leaves of differently aged *Rosa multiflora* seedlings. On April, 2019, the seeds of ‘Mount Shasta’ were sowed in Institute of Vegetables and Flowers, Chinese Academy of Agricultural Sciences (Beijing, China). The leaves were harvested from the plant with 2-3 leaves, and then collected every week until the emergence of buds in seedlings. On June, 2020, the seeds of *Rosa multiflora* were sowed in Meigui Qingyuan Garden (Beijing, China). The leaves were collected for the first time when the plant with 2-3 leaves, and then harvested every month until August, 2021 (not flowering). While the plants have no leaves in winter (2020.11-2021.03), therefore the samples were lacking during the interval. Mean values ± SD are shown from three technical replicates. *RcUBI2* and 5.8s were used as internal controls.


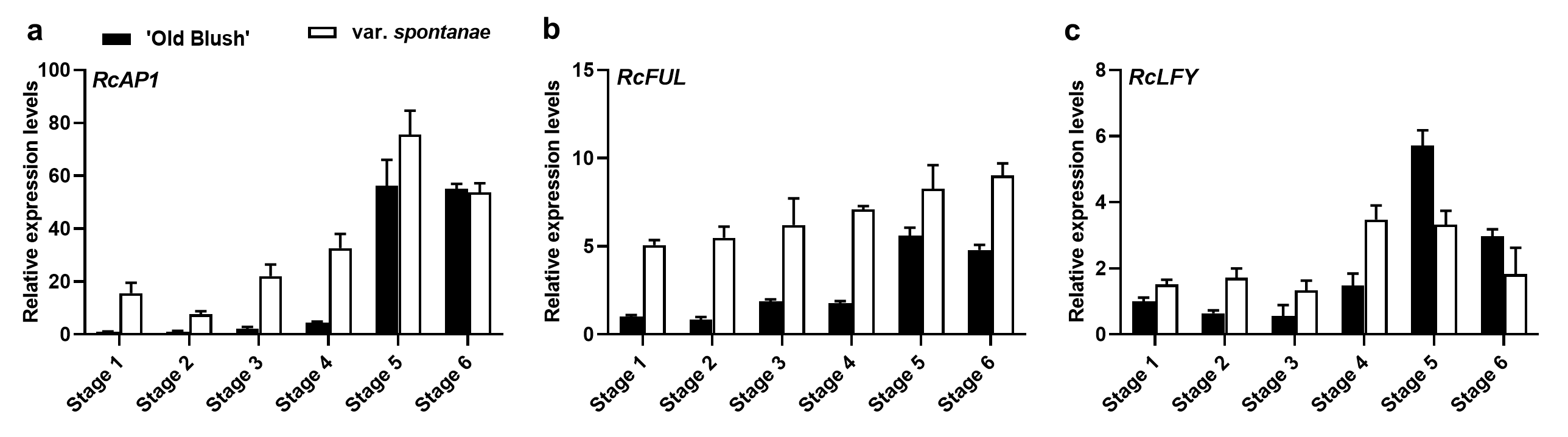


# Supplementary Fig. 8 RT-qPCR analyses of transcript levels of *RcAP1* (a), *RcFUL* (b) and *RcLFY* (c) in ‘Old Blush’ and *R. chinensis* var. *spontanea* during flower development. Stages 1 to 6, flower development stages; stage 1, earliest bud break; stage 2, shoot elongation ( > 20 mm) without 3-leaflet leaf; stage 3, the first 3-leaflet leaf is visible; stage 4, the first 5-leaflet leaf is visible; stage 5, flower bud appearance; stage 6, flower bud with a diameter of 5-7 mm. Mean values ± SD are shown from three technical replicates. *RcUBI2* was used as the internal control.


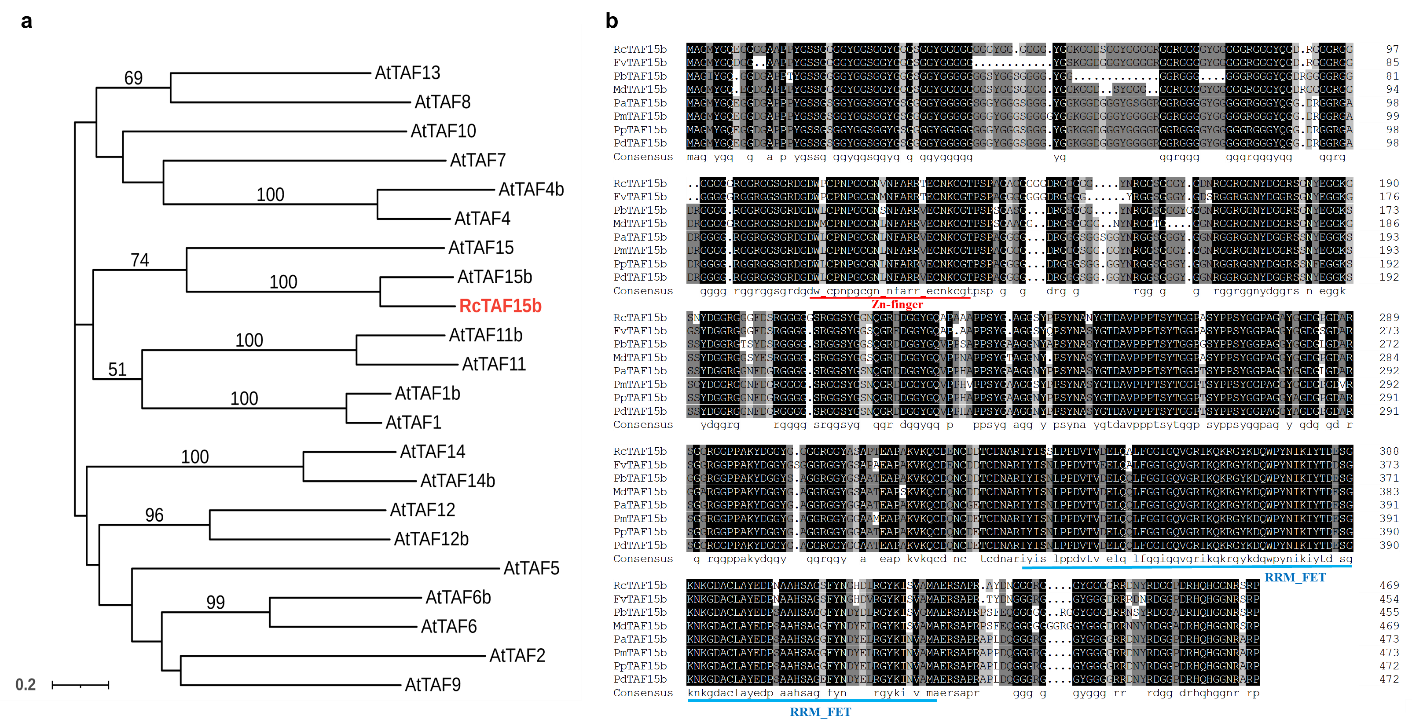


# Supplementary Fig. 9 Bioinformatic analysis of RcTAF15b. (a) Phylogenetic analysis of RcTAF15b and TAF15bs from Arabidopsis. The phylogenetic tree was constructed using MEGA X with neighbor-joining algorithm. Bootstrap values indicate the divergence of each branch, and the scale bare indicates the branch length. AtTAF1 (AT1G32750.1), AtTAF1b (AT3G19040.2), AtTAF2 (AT1G73960), AtTAF4 (AT1G27720), AtTAF4b (AT5G43130), AtTAF5 (AT5G25150), AtTAF6 (AT1G04950), AtTAF6b (AT1G54360), AtTAF7 (AT1G55300), AtTAF8 (AT4G34340), AtTAF9 (At1g54140), AtTAF10 (AT4G31720), AtTAF11 (AT4G20280), AtTAF11b (AT1G20000), AtTAF12 (AT3G10070), AtTAF12b (AT1G17440.1), AtTAF13 (AT1G02680), AtTAF14 (AT2G18000), AtTAF14b (AT5G45600), AtTAF15 (AT1G50300), AtTAF15b (AT5G58470). (b) Alignment analysis of deuced amino acid sequences of RcTAF15b with TAF15b proteins from other plants. The predicted Zn-finger domain and RRM_FET are indicated by red and blue solid lines, respectively. RRM_FET, RNA recognition motif (RRM) found in the FET family of RNA-binding proteins. FvTAF15b (*F. vesca* subsp. *Vesca*, XP_011461457.1), MdTAF15b (*M. domestica*, XP_008355075.2), PaTAF15b (*Prunus avium*, XP_021810524.1), PbTAF1b (*Pyrus* x *bretschneideri*, XP_018505827.1), PdTAF15b (*P. dulcis*, XP_034221551.1), PmTAF15b (*P*. *mume*, XP_008233891.1), PpTAF15b (*P. persica*, XP_020420993.1).


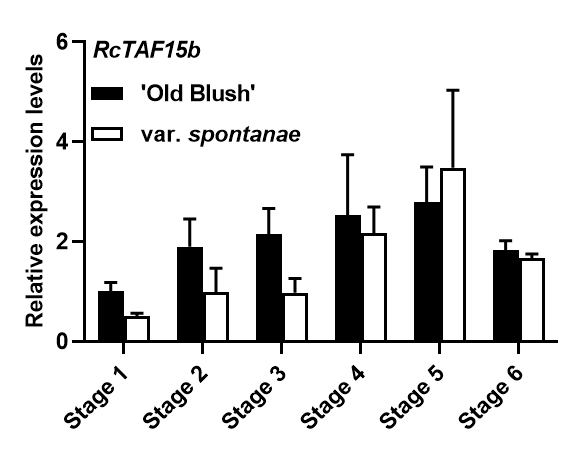


# Supplementary Fig. 10 RT-qPCR analyses of *RcTAF15b* transcript levels in ‘Old Blush’ and *R. chinensis* var. *spontanea* during flower development. Stages 1 to 6, flower development stages; stage 1, earliest bud break; stage 2, shoot elongation ( > 20 mm) without 3-leaflet leaf; stage 3, the first 3-leaflet leaf is visible; stage 4, the first 5-leaflet leaf is visible; stage 5, flower bud appearance; stage 6, flower bud with a diameter of 5-7 mm. Mean values ± SD are shown from three technical replicates. *RcUBI2* was used as an internal control.


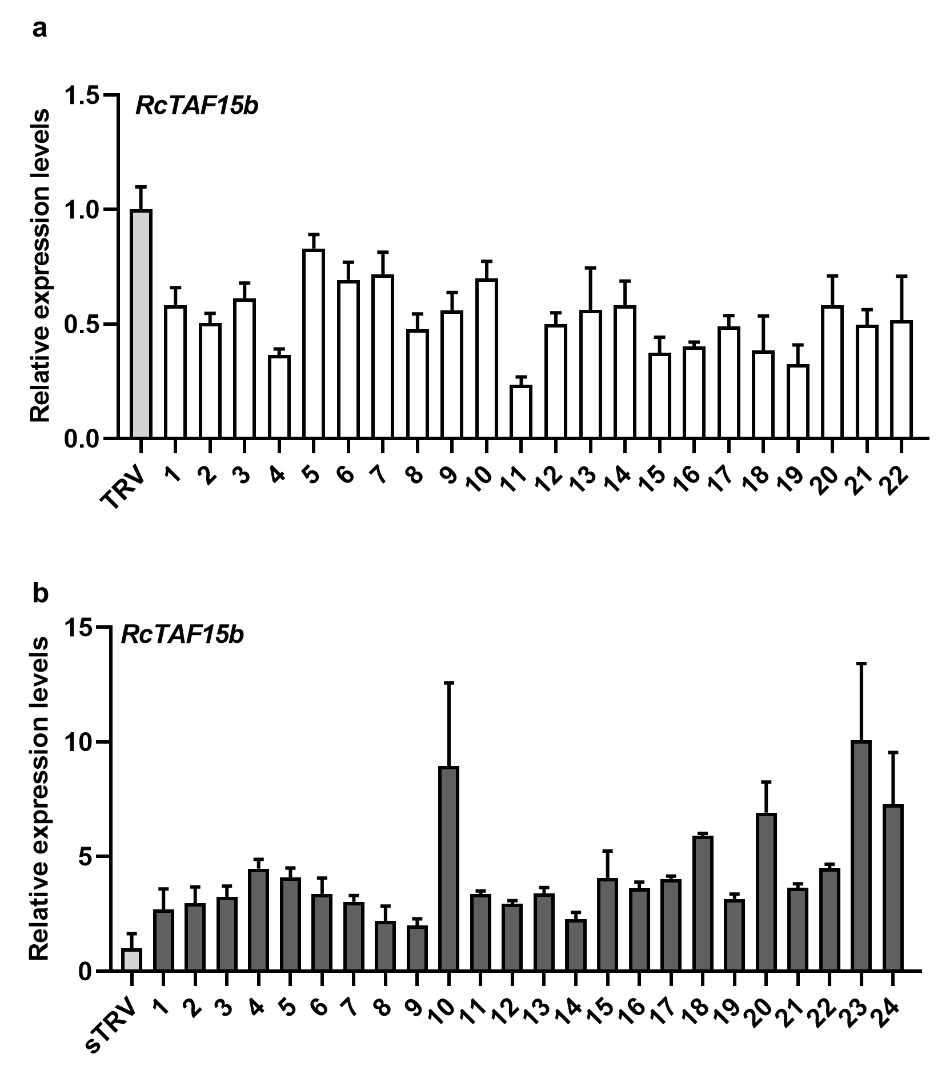


# Supplementary Fig. 11 Transcript levels of *RcTAF15b* in leaves of silenced or overexpressed rose plants. (a) RT-qPCR analysis of *RcTAF15b* expression in TRV control and *RcTAF15b*-silenced (TRV-*RcTAF15b*, No. 1-22) rose plants. *RcUBI2* was used as an internal control. Mean values ± SD are shown from three technical replicates. About 70 plantlets were performed, and about 20 plants with downregulated expression levels of *RcSPL1* were obtained and used for phenotype observation. No. 4, 15 and 16 plants were selected to further RT-qPCR. (b) RT-qPCR analysis of *RcTAF15b* expression in sTRV control and *RcSPL1*-overexpressed (sTRV-*RcTAF15b*, No. 1-24) rose plants. *RcUBI2* was the internal control. Mean values ± SD are shown from three technical replicates. About 70 plantlets were performed, and about 20 plants with upregulated expression levels of *RcTAF15b* were obtained and used for phenotype observation. The No. 3, 7 and 19 plants were selected to further RT-qPCR. VIGS and gene overexpression experiments experiments were performed three times independently, with similar results obtained.

# Supplementary Fig. 12 RT-qPCR analyses of rch-miR156 transcript levels in ‘Old Blush’ and *R. chinensis* var. *spontanea* during flower development. Stages 1 to 6, flower development stages; stage 1, earliest bud break; stage 2, shoot elongation (>20 mm) without 3-leaflet leaf; stage 3, the first 3-leaflet leaf is visible; stage 4, the first 5-leaflet leaf is visible; stage 5, flower bud appearance; stage 6, flower bud with a diameter of 5-7 mm. Mean values ± SD are shown from three technical replicates. *5.8S rRNA* was used as the internal control.

**Supplementary Table 1. Gene ID and information of *SPLs* in the genome database of**

***Rosa. chinensis* 'Old Blush' and *R. chinensis* var. *spontanea***

| **Gene name** | **Gene ID and information in the genome database of *R. chinensis* 'Old Blush'** | **Gene ID and information in the genome database of *R. chinensis* var. *spontanea*** |
| --- | --- | --- |
| *SPL1* | PREDICTED: squamosa promoter-binding-like protein 1 (RchiOBHm_Chr5g0032861) | PREDICTED: squamosa promoter-binding-like protein 1 (evm.model.scaffold379.369) |
| *SPL2* | PREDICTED: squamosa promoter-binding-like protein 2 (RchiOBHm_Chr4g0430121) | PREDICTED: squamosa promoter-binding-like protein 2 (evm.model.scaffold379.332) |
| *SPL3* | PREDICTED: squamosa promoter-binding-like protein 3 (RchiOBHm_Chr5g0008871) | PREDICTED: squamosa promoter-binding-like protein 3 (evm.model.scaffold549.24) |
| *SPL4* | PREDICTED: squamosa promoter-binding-like protein 1 (RchiOBHm_Chr2g0127621) | PREDICTED: squamosa promoter-binding-like protein 4 (evm.model.scaffold584.55) |
| *SPL6* | PREDICTED: squamosa promoter-binding-like protein 6 (RchiOBHm_Chr4g0437871) | PREDICTED: squamosa promoter-binding-like protein 6 (evm.model.scaffold2.50) |
| *SPL7* | PREDICTED: squamosa promoter-binding-like protein 7 (RchiOBHm_Chr4g0420491) | PREDICTED: squamosa promoter-binding-like protein 7 (evm.model.scaffold4637.10) |
| *SPL8* | PREDICTED: squamosa promoter-binding-like protein 8 (RchiOBHm_Chr1g0348581) | PREDICTED: squamosa promoter-binding-like protein 8 (evm.model.scaffold1545.42) |
| *SPL9* | PREDICTED: squamosa promoter-binding-like protein 14 (RchiOBHm_Chr3g0480201) | PREDICTED: squamosa promoter-binding-like protein 9 (evm.model.scaffold70.4) |
| *SPL10* | PREDICTED: squamosa promoter-binding-like protein 7 (RchiOBHm_Chr1g0348611) | PREDICTED: squamosa promoter-binding-like protein 10 (evm.model.scaffold1545.44) |
| *SPL12* | PREDICTED: squamosa promoter-binding-like protein 12 (RchiOBHm_Chr4g0427341) | PREDICTED: squamosa promoter-binding-like protein 12 (evm.model.scaffold885.93) |
| *SPL13A* | PREDICTED: squamosa promoter-binding-like protein 13A (RchiOBHm_Chr7g0189811) | PREDICTED: squamosa promoter-binding-like protein 13A (evm.model.scaffold49.24) |
| *SPL14* | PREDICTED: squamosa promoter-binding-like protein 14 (RchiOBHm_Chr7g0217411) | PREDICTED: squamosa promoter-binding-like protein 14 (evm.model.scaffold470.53) |
| *SPL15* | PREDICTED: squamosa promoter-binding-like protein 16 (RchiOBHm_Chr2g0175901) | PREDICTED: squamosa promoter-binding-like protein 15 (evm.model.scaffold2505.6) |
| *SPL16* | PREDICTED: squamosa promoter-binding-like protein 16 (RchiOBHm_Chr4g0429911) | PREDICTED: squamosa promoter-binding-like protein 16 (evm.model.scaffold379.317) |
| *SPL18* | PREDICTED: squamosa promoter-binding-like protein 6 (RchiOBHm_Chr7g0183471) | PREDICTED: squamosa promoter-binding-like protein 18 evm.model.scaffold509.93 |

Note: The *SPL* genes from the genome database of *R. chinensis* var. *spontanea* (<https://ngdc.cncb.ac.cn/gsa/browse/CRA000589>) were determined via BLASTP with *SPLs* protein sequences from *A. thaliana*, with E-value <1e-5, identity ≥ 50, align_ratio ≥ 0.5*.* A total of 15 SPL family members were identified in *R. chinensis* var. *spontanea* using the SBP-domain profile PF03110 as the search query. The designated names of these genes were according to annotations from the NCBI non-redundant (NR) protein database, the UniProt/Swiss-Prot protein database, and the Kyoto Encyclopedia of Genes and Genomes (KEGG) database. Meanwhile, the corresponding genes in the genome database of ‘Old Blush’ (<https://lipm-browsers.toulouse.inra.fr/pub/RchiOBHm-V2/>) were identified by BLASTN with similar sequences.

**Supplementary Table 2. The different stages of flower development in 'Old Blush' and *R. chinensis* var. *spontanea***

| **Stage** | **External morphology** | **Internal** **morphology** |
| --- | --- | --- |
| 1 | Earliest bud break before elongation | The shoot apical meristem (SAM) is cone-shaped and narrow |
| 2 | Shoot elongation (>20 mm) without 3-leaflet leaf | The meristem is flat-disk shape and enlarges laterally |
| 3 | The first 3-leaflet leaf is visible | The meristem becomes flatter, larger and doming structure |
| 4 | The first 5-leaflet leaf is visible | Sepal and petal primordia are visible |
| 5 | Flower bud appearance but not yet separated | Stamen and carpel primordia initiated and development |
| 6 | Flower bud with a diameter of 5-7 mm. | All floral organs are apparent |

**Supplementary Table 3. Flowering time-related phenotypes of *RcSPL1* overexpression and wild-type *Arabidopsis* plants**

| **Genotype** | **Rosette leaves number at the initiation of abaxial trichome** | **Flowering Days** | **Number of rosette leaves** |
| --- | --- | --- | --- |
| WT | 7.55±0.89 | 31.11±2.22 | 14.57±0.98 |
| *RcSPL1-*OE4 | 6.00±0.86^****^ | 24.29±1.89^**^ | 12.64±1.73^**^ |
| *RcSPL1-*OE14 | 5.65±0.67^****^ | 24.00±2.61^**^ | 12.29±1.50^*^ |
| *RcSPL1-*OE16 | 5.70±0.66^****^ | 22.50±0.58^***^ | 11.57±1.27^**^ |

Note: Plants were grown under long day conditions (16h/8h photoperiod). Genotype: WT, Columbia-0; *RcSPL1-OE*, transgenic lines of *Rc-SPL1*. Indicators of rosette leaves number at the initiation of abaxial trichome, flowering days, and leaf numbers were shown as average ± standard deviation (SD). *, **, *** and **** indicate the significantly different from that of WT at *P* < 0.05, *P* < 0.01, *P* <0.001, *P* <0.0001, respectively.

| **Supplementary Table 4. Proteins putatively interacting with RcSPL1** | | |
| --- | --- | --- |
| **No.** | **Sequence information of NCBI [*Rosa chinensis*]** | **Individual isolations** |
| 1 | PREDICTED: *Rosa chinensis* uncharacterized LOC112166669 (LOC112166669), mRNA | 9 |
| 2 | PREDICTED: *Rosa chinensis* uncharacterized LOC112196371 (LOC112196371), mRNA | 7 |
| 3 | PREDICTED: *Rosa chinensis* transcription initiation factor TFIID subunit 15b (LOC112202151), mRNA | 4 |
| 4 | PREDICTED: *Rosa chinensis* COP9 signalosome complex subunit 5b (LOC112173087), mRNA | 4 |
| 5 | PREDICTED: *Rosa chinensis* vesicle-associated protein 4-1 (LOC112164643), mRNA | 1 |
| 6 | PREDICTED: *Rosa chinensis* elongation factor 1-delta 1 (LOC112195051), mRNA | 1 |
| 7 | PREDICTED: *Rosa chinensis* sm-like protein LSM8 (LOC112182993), mRNA | 1 |
| 8 | PREDICTED: *Rosa chinensis* uncharacterized protein At5g39570 (LOC112186370), mRNA | 1 |

| **Supplementary Table 5. List of primers used** | |
| --- | --- |
| **Primers** | **Sequence (5’→ 3’)** |
| **For quantitative RT-PCR analysis** |  |
| *RcSPL1-*qPCR-F | TTCACTCCAAGGCAGCGATT |
| *RcSPL1*-qPCR-R | TTGATGACTGATACCCCGGC |
| *RcSPL2-*qPCR-F | TCTGTCAACCAGTTCGTGGG |
| *RcSPL2-*qPCR-R | GGTGTTGAGGGATGGCTGTT |
| *RcSPL3-*qPCR-F | AGAGGATGAGGACGAAGAGCA |
| *RcSPL3*-qPCR-R | TAACCTTGTGGCGGCGGTAG |
| *RcSPL4*-qPCR-F | AAGCAGAGGAAGCAGGAGAA |
| *RcSPL4*-qPCR-R | GCACAACTGGAGCCTTAGAAT |
| *RcSPL6*-qPCR-F | GATGTTAAGCGCAGTTGTCG |
| *RcSPL6*-qPCR-R | GTAGAATGCCACCTGGAAGTA |
| *RcSPL7*-qPCR-F | ATTTCCTTTGGCCGTAGGGG |
| *RcSPL7*-qPCR-R | CTCGTCAAACTCCGACACCA |
| *RcSPL8*-qPCR-F | TTTGCCACGAGGATCGGACTT |
| *RcSPL8*-qPCR-R | CGATGACGGTGGAGGCTTTG |
| *RcSPL9*-qPCR-F | AGTTTACAATGTGCTTCGTCT |
| *RcSPL9*-qPCR-R | TACGGCAACTACGTTTCCC |
| *RcSPL10*-qPCR-F | GACGCTCCTCTGCCTCTGCT |
| *RcSPL10*-qPCR-R | AAGCCTTGGAGTGCATCTCA |
| *RcSPL12*-qPCR-F | TGGTCCAACAGTAGTTCCTCT |
| *RcSPL12*-qPCR-R | AGTCCCAACAATCTTCGTCT |
| *RcSPL13A*-qPCR-F | ACTTGAAAGCACCATCTTCGT |
| *RcSPL13A*-qPCR-R | CGTCCACCAACTGATTATTACCT |
| *RcSPL14*-qPCR-F | GCTGGAGCGTAGGACAAGTA |
| *RcSPL14*-qPCR-R | AAGGAGCAACTGAGCCAAT |
| *RcSPL15*-qPCR-F | GAACACCAACAAATTCCTCCCA |
| *RcSPL15*-qPCR-R | GCCCTTGTGCTATGCGGTAA |
| *RcSPL16*-qPCR-F | TCCGACCTTAGCATGTGCAG |
| *RcSPL16*-qPCR-R | TTCCTTCGGCGTTTGTTGTG |
| *RcSPL18*-qPCR-F | CACTCGAAGACCGCCAAAGT |
| *RcSPL18-*qPCR-R | TAGTCCCCGGAAAACTGCTG |
| *RcTAF15b*-qPCR-F | TGCGAGAAGAACTGAGTGTAA |
| *RcTAF15b*-qPCR-R | CTTCATAGTTGCCACTTCGAC |
| *RcAP1-*qPCR-F | CATGAGTCTATGTCCGAACTTCA |
| *RcAP1*-qPCR-R | CCGCTACATTCTTCTCCTTCTC |
| *RcFUL-*qPCR-F | CATCACCACCAGCCCTACTC |
| *RcFUL-*qPCR-R | TGGTGGCATGAGTGTGTTAC |
| *RcLFY-*qPCR-F | GCTATGGATGCTCTCTCTCAAG |
| *RcLFY-*qPCR-R | CTCTCCTAATCCTCCGAAGC |
| *RcUBI2*-qPCR-F | GCCCTGGTGCGTTCCCAACTG |
| *RcUBI2*-qPCR-R | CCTGCGTGTCTGTCCGCATTG |
| *AtAP1*-qPCR-F | GCAGCACCAAATCCAGCATC |
| *AtAP1*-qPCR-R | ACAGACCACCCATGTTGAGA |
| *AtFUL-*qPCR-F | AGAGAAGGTCTGGTTTGCTCA |
| *AtFUL-*qPCR-R | TGCCTTTGGAAGAGAAGACGA |
| *AtLFY-*qPCR-F | TCCCAAGAAGATGATTGGACAGG |
| *AtLFY-*qPCR-R | TGCGTCCCAGTAACCACTTC |
| *AtAcin-*qPCR*-*F | TCTCTATGCCAGTGGTCGTA |
| *AtAcin-*qPCR*-*R | CCTCAGGACAACGGAATC |
| rch-miR156-F | GCGGCGGTGACAGAAGAGAGT |
| 5.8S-F | TGTGACGCCCAGGCAGACG |
| 5.8S-R | CGGCAACGGATATCTCGG |
| U6-qPCR-F | GGAACGATACAGAGAAGATTAGCA |
| Stemloop Universal-R | GTG CAG GGT CCG AGG T |
| U6 cDNA synt | GTG CAG GGT CCG AGG TTT TGG ACC ATT TCT CGA T |
| miR156 stemloop primer | GTCGTATCCAGTGCAGGGTCCGAGGTATTCGCACTGGATACGACGTGCTC |
| **For clone** |  |
| *RcSPL1-*CDS-F | CCAAGTCTTCCCCAACACAC |
| *RcSPL1-*CDS-R | AAAGAAGCCATGGATGACAGAA |
| *RcTAF15b-*CDS-F | TCGCTCTGTTTCTCTCGTTC |
| *RcTAF15b-*CDS-R | CCTTAGCAGCAAGCAATTTGG |
| **For overexpression vector construction** |  |
| *RcSPL1-Xba* Ⅰ-F | TGCTCTAGACCAAGTCTTCCCCAACACAC |
| *RcSPL1-Sac* Ⅰ-R | ACGAGCTCAAAGAAGCCATGGATGACAGAA |
| **For TRV vector construction** |  |
| TRV1-F | TTACAGGTTATTTGGGCTAG |
| TRV1-R | CCGGGTTCAATTCCTTATC |
| TRV2-F | TGGGAGATGATACGCTGTT |
| TRV2-R | CCTAAAACTTCAGACACG |
| *RcSPL1-*VIGS-F | AGGTTACCGAATTCTCTAGACGGGGTATCAGTCATCAATACCG |
| *RcSPL1-*VIGS-R | GTGAGCTCGGTACCGGATCCTGTGATGGCCTATGCTGTTT |
| *RcTAF15b-*VIGS-F | ACCGAATTCTCTAGACAATGCGATGAGAACTGTGA |
| *RcTAF15-*VIGS-R | CTCGGTACCGGATCCGCCATCGCAACACTGATCT |
| **For sTRV vector construction** |  |
| *RcSPL1*-sTRV-F | GGATGAGCTCTACAAAGGTACCATGGAGAGGCAAACTTGG |
| *RcSPL1*-sTRV-R | CGTGAGCTCGGTACCTTAGCTGATCTGGAAATGCT |
| *RcTAF15b*-sTRV-F | GGATGAGCTCTACAAAGGTACCATGGCTGGGATGTATGGT |
| *RcTAF15b*-sTRV-R | CGTGAGCTCGGTACCTCAATATGGACGTGAACGGT |
| **For subcellular localization** |  |
| pSuper1300-*RcSPL1*-GFP-F | AGTCTAGAAAGCTTATGGAGAGGCAAACTTGGAAA |
| pSuper1300-*RcSPL1*-GFP-R | CCCTTGCTCACCATGGTACCGCTGATCTGGAAATG |
| **For miRNA regulation assay** |  |
| Pri-rch-miR156-F | ATGAGAGGGAATGAAGAGAG |
| Pri-rch-miR156-R | GGAACGAACTGGGTAGGTGT |
| *RcSPL1*-sensor-GFP-F | GACTCTAGTCTAGATAGCTCCCTCTCTTCTGTCAGGTACCATGGTGAGCA |
| *RcSPL1*-sensor-GFP-R | TGCTCACCATGGTACCTGACAGAAGAGAGGGAGCTATCTAGACTAGAGTC |
| His-*RcSPL1*-F | tctagcggcgagctcATGGAGAGGCAAACTTGGAA |
| His-*RcSPL1*-R | gggaaattcgagctcTTAGCTGATCTGGAAATGCT |
| His-*RcSPL1-3′UTR*-F | tctagcggcgagctcATGGAGAGGCAAACTTGGAA |
| His-*RcSPL1-3′UTR*-R | cgatcggggaaattcgagctcTTACCCTATTAACA |
| **For yeast two-hybrid system** |  |
| pGBKT7-*RcSPL1-*F | CAGAGGAGGACCTGCATATGATGGAGAGGCAAACTTGGAA |
| pGBKT7-*RcSPL1*-R | CGCTGCAGGTCGACGGATCCTTAGCTGATCTGGAAATGCT |
| pGADT7-*RcTAF15b*-F | TGGAGGCCAGTGAATTCATGGCTGGGATGTATGGTCA |
| pGADT7-*RcTAF15b*-R | GAGCTCGATGGATCCTCAATATGGACGTGAACGGTTT |
| **For BiFC assay** |  |
| SPYCE-*RcTAF15b*-F | GCCACTAGTGGATCCATGGCTGGGATGTATGGTCA |
| SPYCE-*RcTAF15b*-R | AGCGGTACCCTCGAGATATGGACGTGAACGGTTTC |
| SPYNE-*RcSPL1*-F | CCTACTAGTGGATCCATGGAGAGGCAAACTTGGAA |
| SPYNE-*RcSPL1*-R | AGCGGTACCCTCGAGGCTGATCTGGAAATGCTTGG |
| **For Co-localization assay** |  |
| 130-*RcTAF15b*-mCherry-F | ACGGGGGACTCTAGAATGGCTGGGATGTATGGTCA |
| 130-*RcTAF15b*-mCherry-R | CATCCCGGGTACCGGATATGGACGTGAACGGTTTC |
